# Impacts of Surface Characteristics and Dew Point on the Blue-Light (BL_405_) Inactivation of Viruses

**DOI:** 10.3390/microorganisms11112638

**Published:** 2023-10-26

**Authors:** Castine Bernardy, James Malley

**Affiliations:** Department of Civil and Environmental Engineering, College of Engineering & Physical Sciences, University of New Hampshire, Durham, NH 03824, USA; castine.bernardy@unh.edu

**Keywords:** BL_405_, blue light, surface, dew point, inactivation, MS2, dose–response, ceramic, PTFE, stainless steel

## Abstract

The increased prevalence of multidrug-resistant organisms (MDROs), healthcare associated infections (HAIs), and the recent COVID-19 pandemic has caused the photoinactivation industry to explore alternative wavelengths. Blue light (BL_405_) has gained significant interest as it is much less harmful to the skin and eyes than traditional germicidal wavelengths; therefore, in theory, it can be used continuously with human exposure. At present, the viricidal effects of BL_405_ are largely unknown as the literature predominately addresses bacterial disinfection performed with this wavelength. This work provides novel findings to the industry, reporting on the virucidal effects of BL_405_ on surfaces. This research utilizes three surfaces: ceramic, PTFE, and stainless steel. The efficacy of BL_405_ inactivation varied by surface type, which was due to surface characteristics, such as the contact angle, porosity, zeta potential, and reflectivity. Additionally, the effect of the dew point on BL_405_ inactivation efficacy was determined. This research is the first to study the effects of the dew point on the virucidal effectiveness of BL_405_ surface inactivation. The effects of the dew point were significant for all surfaces and the control experiments. The high-dew-point conditions (18 °C) yielded higher levels of BL_405_ inactivation and viral degradation for the experiments and controls, respectively.

## 1. Introduction

The COVID-19 pandemic highlighted the need for more robust surface disinfection techniques. The germicidal properties of UV_254_ are well understood [1,2,3], and new developments in the photonics industry suggest that other wavelengths may have germicidal effects [4]. Visible light, specifically blue light at 405 nm, is a promising new disinfection technology [5,6].

The healthcare industry has taken an interest in the germicidal properties of blue light, as hospitals continue to struggle with millions of cases of hospital-associated infections (HAIs) [7]. These technologies can provide another barrier of protection for hospital patients and may also assist in addressing the rising threat of multidrug-resistant organisms (MDROs) [8]. Additionally, exposure to 405 nm is considered safe for human contact [5]; therefore, blue-light technologies have the potential to provide a continuous low level of disinfection.

Unlike UV wavelengths, which are known to cause erythema and photokeratitis [2,9,10], this visible-light wavelength is considered safe for human skin and eye exposure [11]. The 405 nm range does not share the harmful effects of BL_440_ and BL_480,_ which are known to cause photo retinitis and the disruption of the circadian rhythm, respectively [5].

The relative safety of blue light was compared to the traditional wavelength, UV_254_, by the American Conference of Governmental Industrial Hygienists [12]. Their 2021 publication released threshold limit values (TLVs) at wavelengths between 180–400 nm. The TLV for 405 nm was extrapolated from the data and compared to 254 nm. The TLVs set for 254 and 405 nm were 6.0 mJ/cm^2^ and 1.2 × 10^5^ mJ/cm^2^, respectively. Exposures up to these TLVs are considered safe for human eyes and skin, without experiencing complications due to erythema and photokeratitis. The TLV value for 405 nm is five orders of magnitude higher than the TLV for 254 nm, providing sufficient evidence that 405 nm wavelengths are safer for human skin and eye exposure than 254 nm [12]. BL_405_ is particularly advantageous as this wavelength can provide a continuous, low level of disinfection, as exposure to people at doses lower than the TLV in public places is not a health concern.

Applications of BL_405_ disinfection in the literature [5,13,14,15] predominantly focus on bacterial and fungal studies. There is a consensus that the BL_405_ inactivation of bacteria and fungi is caused by interactions with porphyrin molecules within the fungal or bacterial cells. The blue-light wavelength is absorbed by the porphyrin molecules, which become photo excited. The photo-excited porphyrins then react with other cellular components or oxygen, leading to the creation of reactive oxygen species (ROS). The ROS leads directly to cell death via oxidative damage to the cell membrane (proteins and lipids) [5,13,14,15]. ROS-induced cell damage occurs via two pathways: type 1, the creation of superoxide ions via electron transfer, or type 2, the production of singlet oxygen. Both pathways cause sufficient damage to the cellular components and ultimately lead to cell death [16].

Studies on the effectiveness of 405 nm technologies for viral inactivation are limited. It was previously thought that, due to the lack of porphyrins in viruses, this technology would not be effective for viruses without the use of an added photosensitizer. The industry calls for more research on the viricidal effects of blue light to determine its effectiveness and potential applications [13,14].

Previous work [17,18,19,20] has revealed that surface type and characteristics greatly influence the efficacy of photoinactivation of the virus. Factors, such as shadowing and porosity, were studied by Moore et al. [18] and Tomas et al. [20], respectively. Bernardy et al. [19] studied UV_254_ inactivation efficacy on five surface types: aluminum, ceramic, Formica laminate, PTFE, and stainless steel. Their work investigated the effects of viral recovery from these surfaces as a function of the contact angle, surface roughness, porosity, reflectivity, and zeta potential. These results reinforce that surface type/characteristics greatly impact the efficacy of line-of-sight inactivation technologies, such as UV_254_ or BL_405_.

The effects of ambient environmental conditions on the photoinactivation of viruses have been studied in the literature [2,21,22] for UV_254_, which may provide some insights into the effects on BL_405_. Kowalski et al. [2] tabulated UV_254_ viral inactivation data at varying relative humidities. These findings revealed that the effect of relative humidity on viral species was largely species dependent and found that general conclusions regarding the effect of relative humidity on the UV_254_ inactivation of viruses could not be assessed.

However, the research exploring the effect of temperature, relative humidity, or dew point have not been conducted previously with BL_405_ technologies. Therefore, in this paper, the data are developed to elucidate the effects of high and low-dew-point conditions, which consider the interaction of temperature and relative humidity, for the BL_405_ inactivation of the virus.

The objective of this study is to address the many research gaps in the literature on BL_405_ inactivation. This work enhances the body of knowledge of BL_405_ viricidal effects. The effects of surface characteristics are explored using ceramic, polytetrafluoroethylene (PTFE), and stainless-steel disks. The inactivation of MS2 bacteriophage, a +ssRNA (positive-sense single-stranded ribonucleic acid) virus commonly studied in photoinactivation research, is tested on these surfaces. The experiments were repeated at high (18 °C) and low (−4 °C) dew points to also determine if these environmental conditions had an impact on the BL_405_ inactivation efficacy. This novel work explores the viricidal impacts of the surface characteristics and dew point for BL_405_ inactivation applications, which have not been studied in the literature. This research provides significant contributions to the BL_405_ inactivation industry, which will ultimately lead to the greater protection of the public’s health.

## 2. Materials and Methods

### 2.1. Surfaces

The surfaces evaluated for this work included ceramic, PTFE, and stainless steel. Table 1 below displays the details of each surface type utilized in the experiments.

Table 1 displays the specifications and manufacturer information for each of the materials utilized for the experimentation.

The surface porosity, zeta potential, and contact angle of these surfaces were quantified. The exact methods used to quantify these characteristics were described in a previous publication (Bernardy et al.) [19].

### 2.2. Experimental Conditions

This research utilized a controlled environmental chamber to conduct the experiments. Table 2 below displays the temperature and relative humidity conditions in the controlled chamber.

Table 2 displays the dew-point conditions maintained in the experimental chamber.

These dew-point conditions were selected as they were representative of the average annual high and low dew points in the United States reported by the National Oceanic and Atmospheric Administration [23]. The relative humidity and temperature were carefully monitored during the experimentation using EXTECH Instruments, Humidity Alert II. The environmental chamber controlled the temperature, while humidifiers and dehumidifiers controlled the relative humidity.

### 2.3. BL_405_ Irradiance and Dose

To determine the range of BL_405_ doses needed for this work, a literature review was conducted. The results of the review were tabulated and are shown in the Supplementary Materials (Figure S1). The characteristics of each virus studied in the literature [14,24,25,26] were compared to the characteristics of the MS2 bacteriophage surrogate used in this study and are shown in Table S1. The doses selected for experimentation were 0, 50, 100, and 200 J/cm^2^, based on the data from viruses that were structurally similar to MS2.

**Figure 1:**
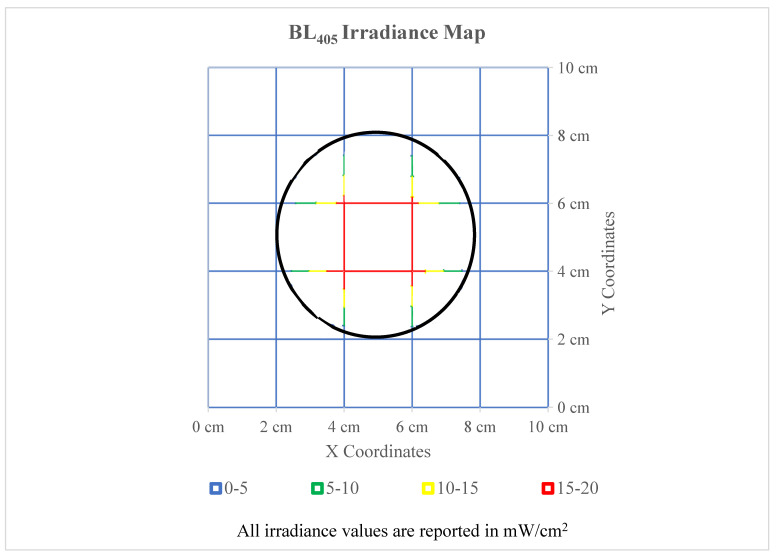
BL_405_ irradiance map emitted by the Thorlabs collimated beam. The collimated beam was positioned 34 cm above the radiometer for these measurements. The weighted average irradiance at this height was 13 mW/cm^2^ inside of the black circle, which represents the outline of the surfaces.

These experiments utilized a collimated LED manufactured by Thorlabs, model M405L4C1—405 nm. To accurately quantify the BL_405_ dose, the average irradiance received by the surfaces was determined using similar methods to those of Linden and Bolton [27]. A 10 by 10 cm grid was created and the irradiance values were recorded with a calibrated NIST traceable IL1700 radiometer every 2 cm in the X and Y directions. The radiometer was calibrated in compliance with National Institute of Standards and Technology (NIST) practices recommended in the NIST Handbook 150-2E [28]. Figure 1 displays the spread of irradiance emitted by the Thorlabs collimated beam unit and was used to determine a weighted average of the irradiance that the surfaces received.

The average irradiance received by the surfaces (13 mW/cm^2^) was determined by the black outline displayed in the irradiance map shown in Figure 1. The collimated beam was positioned 34 cm above the radiometer, as this height produced the most even spread of irradiance.

To determine the BL_405_ doses received during the experimentation, the following equation was utilized:

BL_405_ Dose (mJ/cm^2^) = BL_405_ Irradiance (mW/cm^2^) × Surface Exposure Time (Seconds)

The corresponding surface exposure times were determined by dividing the target BL_405_ dose by the average irradiance value and are shown in Table 3.

Table 3 displays the blue-light doses and corresponding exposure times used for the experimentation.

The BL_405_ doses were delivered using the times shown in Table 3 and corresponded to 0, 50, 100, and 200 J/cm^2^. These were referred to as the ‘BL_405_ Light On’ data points. Additionally, due to the long times required to achieve adequate doses of BL_405_, the effect of viral degradation over time was quantified for each surface using control runs that were referred to as ‘405 Light Off’ data points.

### 2.4. Rate Constants (k Values)

Two rate constants (k) were determined for each surface: non-viricidal and viricidal. These constants represented the viricidal effects of BL_405_ and viral degradation (non-viricidal) due to the long exposure times of these experiments. The k values were calculated from polynomial equations, as they helped to illustrate the different regions of the dose–response curves (shoulder, exponential, and tailing), which was consistent with the industry’s guidance [29]. The EPA does not advise the use of extrapolation from these equations outside of the tested dose range. Mattle et al. [30] studied the effects of tailing during the UV_254_ inactivation of MS2 bacteriophage. The authors found that after exponential decay, the inactivation rate decreased due to clumping (aggregation) and recombination. Bernardy et al. [19] suggested that polynomial fits were the best to incorporate the effects of the surface characteristics on the dose–response curve. Furthermore, readers are cautioned against extrapolating outside of the tested data range.

#### 2.4.1. Non-Viricidal Rate Constants

The non-viricidal rate constants were calculated to determine the viral degradation over time at the high- and low-dew-point conditions. These values represent the experiments conducted without turning on the BL_405_ collimated beam and are shown in the dose response curved as ‘405 Light Off’ data points. The slope of the line at each interval was used to find the k values. These values were averaged to find the rate of change of the polynomial.

#### 2.4.2. Viricidal Rate Constants

The viricidal rate constant was calculated using similar methods as described for the time exposure-rate constants. The viricidal rate constant represents the true viricidal effect of BL_405_ on MS2 by surface type and dew point. This rate constant (k value) excluded the losses due to viral degradation over time to achieve an understanding of the effects solely due to BL_405_ inactivation.

The equation to determine the viricidal k value is shown below:

Viricidal k (1/h) = Total Loss k (1/h) − Viral Degradation k (1/h)

The viricidal k value was converted to units of 1/s and then divided by the BL_405_ irradiance value (W/cm^2^) to produce a k value with units that were consistent with the literature, cm^2^/J.

### 2.5. MS2 Bacteriophage

The surrogate used for experimentation was the MS2 bacteriophage. For detailed information regarding the contract laboratory used for the analysis of the MS2 samples, please refer to a previous publication [19].

### 2.6. Experimental Procedure

The surfaces were inoculated with the MS2 bacteriophage using a cotton swab. The swab was submerged in a stock MS2 solution and then applied to the respective surface. A 5 cm-diameter stencil was placed over the surfaces prior to swabbing to keep the inoculation area consistent between the surfaces. This method provided the most reproducible results of all the inoculation methods tested. Previous methods that were tested included using a spray bottle and nebulizer.

After receiving their respective BL_405_ doses, the surfaces were rinsed with 50 mL of sterile phosphate-buffered solution (PBS) using a pointed spray nozzle. A clamp was used to hold the surface above a beaker, which collected the PBS rinse water. Each experiment (surface type and dew-point condition) was run in triplicate. The samples of PBS rinse water were sent to a contracted lab (GAPLAB, London, ON, Canada) for a plaque assay analysis to determine the remaining MS2 bacteriophage infectivity for each experimental condition. The contracted lab split the samples into duplicates for QA/QC purposes; therefore, each blue-light dose had a total of six data points.

All materials used in the laboratory were sterilized to prevent contamination. The stainless steel, glassware, and phosphate-buffered solution were autoclaved prior to experimentation. The ceramic, PTFE, plastic stencils, and clamps were disinfected in a bleach solution. These materials received a 20 min contact time in the bleach solution, after which they were thoroughly rinsed with tap water, followed by RO water. These items were then set out to dry before the subsequent experiment.

Negative-control experiments were conducted in our laboratory on bleached and autoclaved materials. The MS2 bacteriophage recovery was below the detection limit for all the materials tested. These data can be found in the compiled data DOI listed at the bottom of this manuscript. In addition, MS2 concentration checks were performed every experimental day to ensure the health of the viral stock prior to analysis.

### 2.7. Statistical Analysis

Statistical analyses were conducted to determine if the surface type and dew point significantly affected BL_405_ inactivation and viral degradation over time. These tests were conducted using the Fit Model platform in JMP, version 16.1. The effects with *p*-values less than 0.05 were considered as significant. For more information, refer to the Supplementary Materials Section.

## 3. Results

**Figure 2:**
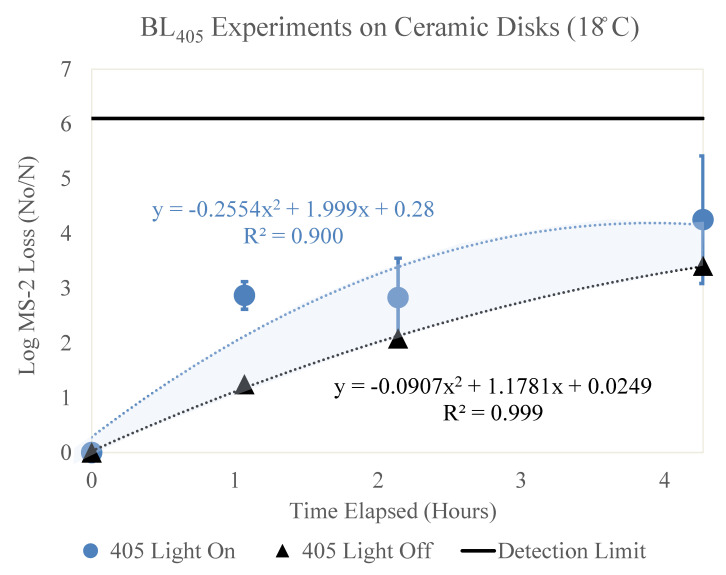
Log loss of MS2 bacteriophage as a function of time on ceramic disks in a high-dew-point environment. Time in hours is shown on the x axis and log loss of MS2 is shown on the y axis. Two trendlines are displayed, indicating the log loss of MS2 as a function of time with and without exposure to BL_405_ irradiance. The shaded region between the two trendlines represents the viricidal effect of BL_405_ on ceramic disks in high-dew-point environments. The microbial detection limit for these experiments is shown as a line at the top of the figure.

**Figure 3:**
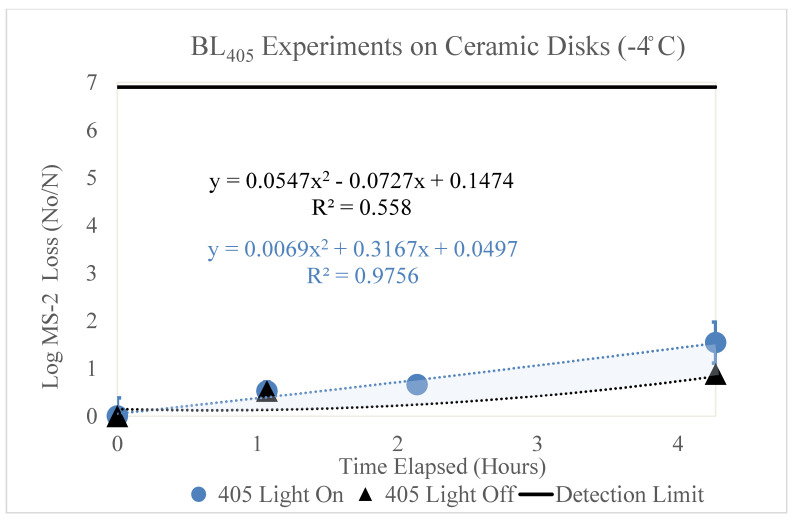
Log loss of the MS2 bacteriophage as a function of time on ceramic disks in a low-dew-point environment. Time in hours is shown on the x axis and the log loss of MS2 is shown on the y axis. Two trendlines are displayed, indicating the log loss of MS2 as a function of time with and without exposure to BL_405_ irradiance. The shaded region between the two trendlines represents the viricidal effect of BL_405_ on ceramic disks in low-dew-point environments. The microbial detection limit for these experiments is shown as a line at the top of the figure.

**Figure 4:**
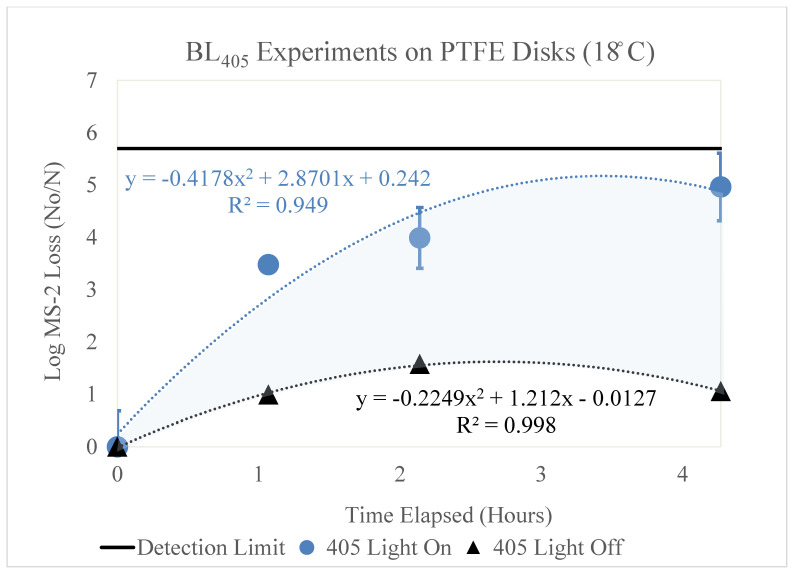
Log loss of the MS2 bacteriophage as a function of time on PTFE disks in a high-dew-point environment. Time in hours is shown on the x axis and the log loss of MS2 is shown on the y axis. Two trendlines are displayed, indicating the log loss of MS2 as a function of time with and without exposure to BL_405_ irradiance. The shaded region between the two trendlines represents the viricidal effect of BL_405_ on PTFE disks in high-dew-point environments. The microbial detection limit for these experiments is shown as a line at the top of the figure.

**Figure 5:**
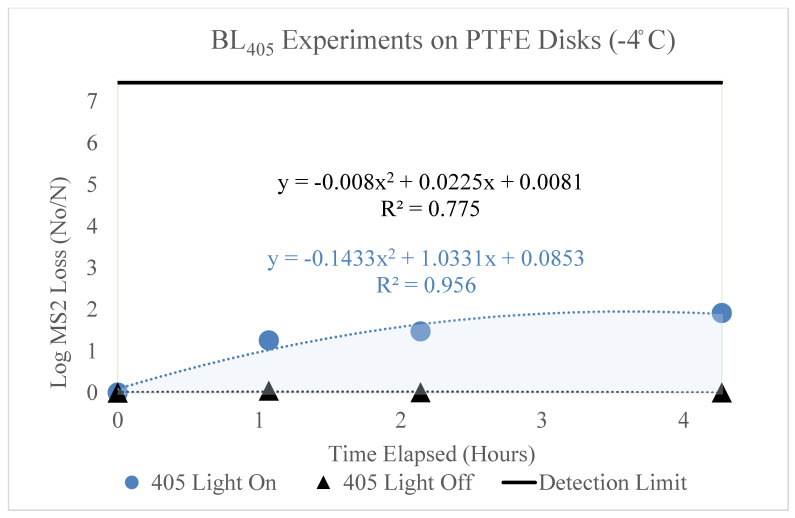
Log loss of MS2 bacteriophage as a function of time on PTFE disks in a low-dew-point environment. Time in hours is shown on the x axis and the log loss of MS2 is shown on the y axis. Two trendlines are displayed, indicating the log loss of MS2 as a function of time with and without exposure to BL_405_ irradiance. The shaded region between the two trendlines represents the viricidal effect of BL_405_ on PTFE disks in low-dew-point environments. The microbial detection limit for these experiments is shown as a line at the top of the figure.

**Figure 6:**
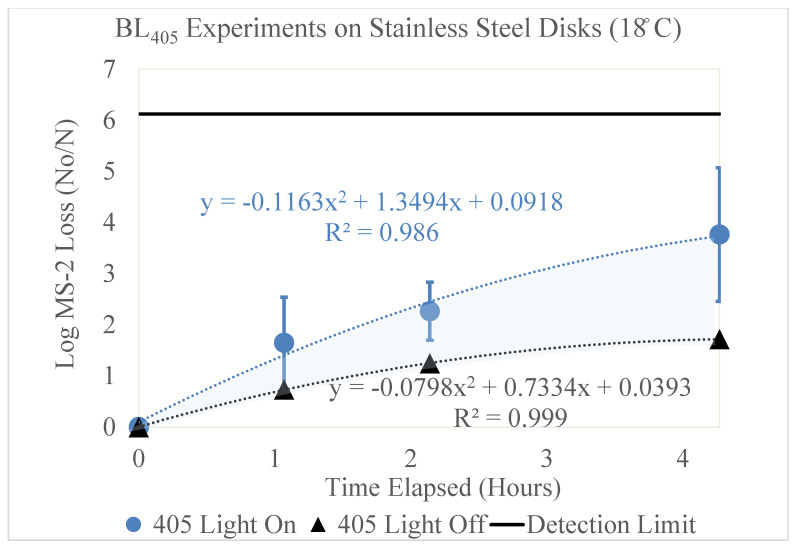
Log loss of MS2 bacteriophage as a function of time on stainless-steel disks in a high-dew-point environment. Time in hours is shown on the x axis and the log loss of MS2 is shown on the y axis. Two trendlines are displayed, indicating the log loss of MS2 as a function of time with and without exposure to BL_405_ irradiance. The shaded region between the two trendlines represents the viricidal effect of BL_405_ on the stainless-steel disks in high-dew-point environments. The microbial detection limit for these experiments is shown as a line at the top of the figure.

Figure 2, Figure 3, Figure 4, Figure 5 and Figure 6 display the BL_405_ inactivation data for the MS2 bacteriophage on ceramic, PTFE, and stainless-steel surfaces. The results show the effect of blue-light inactivation at high (18 °C) and low (−4 °C) dew points on these three surfaces. These figures are displayed as a function of time. Two trendlines exist, representing the log loss of MS2 with the BL_405_ on and off.

The results of the ceramic high- and low-dew-point experiments are shown below.

### 3.1. Ceramic

#### 3.1.1. High Dew Point (18 °C)

Figure 2 displays the log MS2 bacteriophage loss as a function of time (hours). Two trendlines are shown, representing the loss of MS2 with (blue circle) and without (black triangle) exposure to BL_405_ irradiance. The BL_405_ doses corresponding to the time (hours) shown for the ‘405 Light On’ data are 0, 50, 100, and 200 J/cm^2^, respectively. Three trials were conducted for each BL_405_ dose; the figure displays the average of these trials and the standard deviation as error bars.

Additionally, the figure displays the MS2 degradation as a function of time without exposure to BL_405_ irradiance. These data indicate the significant effect of time, with an increase in the log loss as the elapsed time increased.

Both trendlines were adjusted to account for the retention of MS2 on the ceramic disks. The area between these two trendlines (shaded blue) represents the viricidal effect of BL_405_. This area excludes any losses due to viral degradation on the surface.

#### 3.1.2. Low Dew Point (−4 °C)

Figure 3 displays the log MS2 bacteriophage loss as function of time (hours). Two trendlines are shown, representing the loss of MS2 with (blue circle) and without (black triangle) exposure to BL_405_ irradiance in a low-dew-point environment. The BL_405_ doses corresponding to the time (hours) shown for the ‘405 Light On’ data are 0, 50, 100, and 200 J/cm^2^, respectively. Three trials were conducted for each BL_405_ dose; the figure displays the average of these trials and the standard deviation as error bars. When compared to the high-dew-point data, the effect of BL_405_ was reduced in the low-dew-point experimental environment.

Additionally, the black trendline displays the log loss of MS2 as function of time, without exposure to BL_405_ irradiance. The effect of viral degradation over time on the ceramic disks was less in the low-dew-point environment compared to the high-dew-point environment.

Both trendlines were adjusted to account for the retention of MS2 on the ceramic disks. The area between these two trendlines (shaded blue) represents the viricidal effect of BL_405_. This area excludes any losses due viral degradation over time on the surface. To determine the viricidal effects of BL_405_ without the confounding effects of viral degradation over time, the k value for the ‘405 Light Off’ was subtracted from the ‘405 Light On’ k value. These data are shown in Table 4.

The viricidal k value for BL_405_ in high-dew-point environments was slightly higher than the viricidal k value in low-dew-point environments. The high-dew-point experiments experienced a higher log loss of MS2 at each BL_405_ dose, when compared to the low-dew-point experiments. The viral loss over time was much higher at the high dew point; therefore, the true viricidal effects of BL_405_ in each environmental condition were similar.

### 3.2. PTFE

The results of the high- and low-dew-point experiments on PTFE disks are shown below.

#### 3.2.1. High Dew Point

Figure 4 displays the log MS2 bacteriophage loss as a function of time (hours). Two trendlines are shown, representing the loss of MS2 with (blue circle) and without (black triangle) exposure to BL_405_ irradiance. The irradiance corresponding to the ‘405 Light On’ trendline was 13 mW/cm^2^; therefore, it is representative of BL_405_ doses of 0, 50, 100, and 200 J/cm^2^. Three trials were conducted for each BL_405_ dose; the figure displays the average of these trials and the standard deviation as error bars. The highest level of inactivation (4.96 log) was achieved at a BL_405_ dose of 200 J/cm^2^.

Additionally, the figure displays the MS2 loss as a function of time without exposure to BL_405_ irradiance. The log MS2 degradation varied over time, plateauing after approximately 2 h. The peak degradation of MS2 was 1.5 log for these time exposure experiments.

Both trendlines were adjusted to account for the retention of MS2 on the PTFE disks. The area between these two trendlines (shaded blue) represents the viricidal effect of BL_405_. This area excludes any losses due to viral degradation over time on the surface.

#### 3.2.2. Low Dew Point (−4 °C)

The loss of the MS2 bacteriophage over time (hours) on PTFE disks is shown in Figure 5. This figure displays two trendlines, representing the loss of MS2 with (blue circle) and without (black triangle) exposure to BL_405_ irradiance. The irradiance corresponding to the ‘405 Light On’ trendline was 13 mW/cm^2^; therefore, it is representative of BL_405_ doses of 0, 50, 100, and 200 J/cm^2^. Three trials were conducted for each BL_405_ dose; the figure displays the average of these trials and the standard deviation as error bars. The highest level of inactivation (1.91 log) was achieved at a BL_405_ dose of 200 J/cm^2^. The ‘405 Light On’ data points displayed very little variation; therefore, the error bars on the trendline were hidden.

Additionally, the figure displays the MS2 degradation as a function of time without exposure to BL_405_ irradiance. It appears that viral degradation was not significant over time. This trendline displays an essentially flat line, indicating no effect of time.

Both trendlines were adjusted to account for the retention of MS2 on the PTFE disks. The area between these two trendlines (shaded blue) represents the viricidal effect of BL_405_. This area excludes any losses due to viral degradation on the surface.

BL_405_ was more effective on the PTFE surfaces in the high-dew-point conditions. Although the high dew point yielded greater MS2 loss over time, the total loss (‘405 Light On’) was significantly higher than the total loss for the low-dew-point experiments. Essentially, no viral degradation due to time exposure was observed in the low-dew-point conditions on the PTFE surface.

### 3.3. Stainless Steel

The results of the high-dew-point experimental conditions for the stainless-steel disks are shown in Figure 6.

#### High Dew Point (18 °C)

Figure 6 displays the log loss of the MS2 bacteriophage as a function of time, measured in hours. This figure shows two trendlines, representing the loss of MS2 with (blue circle) and without (black triangle) exposure to BL_405_ irradiance. The BL_405_ irradiance corresponding to the ‘405 Light On’ trendline was 13 mW/cm^2^; therefore, it is representative of BL_405_ doses of 0, 50, 100, and 200 J/cm^2^. Three trials were conducted for each BL_405_ dose; Figure 6 displays the average of these trials and the standard deviation as error bars. The highest level of inactivation on the stainless-steel disks (3.76 log) was achieved at a BL_405_ dose of 200 J/cm^2^.

Additionally, the black trendline displays the effect of time on MS2 degradation. This represents experiments conducted without exposure to BL_405_ irradiance, measuring the effects of viral degradation over time. As the time increased, the viral degradation increased. The highest viral loss, 1.72 log, was observed after 4.3 h.

Both trendlines were adjusted to account for the viral retention on the stainless-steel disks. The area between these two trendlines (shaded blue) represents the viricidal effect of BL_405_. This area excludes any losses due to viral degradation on the surface.

These experiments were repeated on the stainless-steel disks in the low-dew-point environment. No significant effect of BL_405_ inactivation occurred on the stainless-steel disks in low-dew-point conditions; this is displayed in the Supplementary Materials (Figure S2).

### 3.4. k Values for Each Surface by Dew Point

Figure 2, Figure 3, Figure 4, Figure 5 and Figure 6 show that BL_405_ inactivation efficacy and viral degradation over time are affected by surface type and dew point. The viricidal k values from each surface type and dew-point conditions were tabulated and are shown in Table 4. The values were calculated by subtracting the total loss of ‘405 Light On’ from the ‘405 Light Off’ k values for the true viricidal k values. The k values from the trendlines were divided by the average irradiance to report units of cm^2^/J.

**Table 4:** Viricidal k values for each surface by dew point.

Surface	High-Dew-Point k (cm^2^/J)	Low-Dew-Point k (cm^2^/J)
Ceramic	0.0093	0.0087
PTFE	0.0385	0.0210
Stainless steel	0.0193	No Data

Table 4 displays the viricidal k values for each surface at high and low dew points.

As shown in Table 4, the high-dew-point k values (cm^2^/J) are higher than the low-dew-point k values, indicating that BL_405_ inactivation is greater in the high-dew-point environment. The data for the stainless steel at the low dew point was inconclusive, therefore was not included in this data set. The PTFE surfaces achieved the highest level of inactivation at both dew-point conditions, whereas the ceramic surfaces exhibited the lowest.

Figure 2, Figure 3, Figure 4, Figure 5 and Figure 6 demonstrate a trend where viral degradation increases in the high-dew-point conditions for each surface type. Table 5 displays the variation, or increase, in viral degradation in the high-dew-point environment, compared to the low dew point environment. The viral degradation rates are shown as a function of time (1/h). These data were tabulated from the ‘405 Light Off’ trendlines shown in Figure 2, Figure 3, Figure 4, Figure 5 and Figure 6 and are displayed in Table 5 as non-viricidal k values.

Table 5 displays the non-viricidal k values for each surface at the high- and low-dew-point conditions.

**Figure 7:**
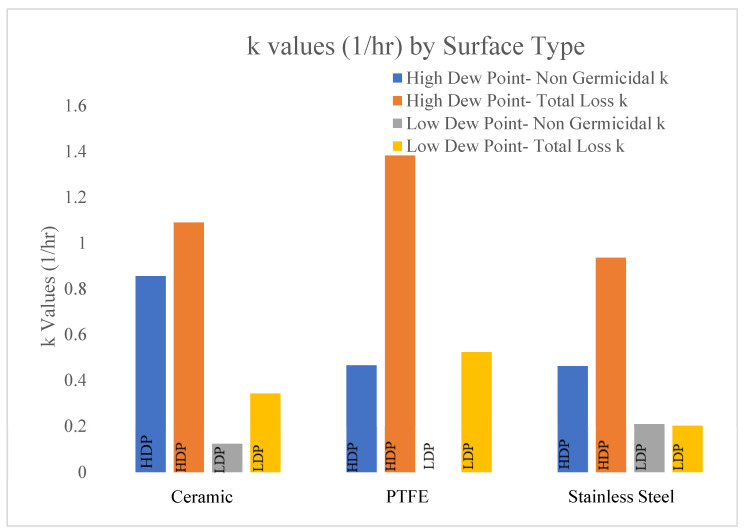
Comparison of the total loss (405 ‘Light On’) and non-viricidal k values (405 ‘Light Off’) for each surface type. The surface type and dew point are on the x axis. The k values (1/h) are shown on the y axis. The blue and orange bars represent the non-viricidal and total losses, respectively, for each surface in the high-dew-point conditions. The gray and yellow bars represent the non-viricidal and total losses, respectively, in the low-dew-point conditions.

As shown in Table 5, the non-viricidal k values are higher for each surface in the high-dew-point conditions compared to the low-dew-point conditions. These data are shown in Figure 7. Figure 7 displays the total loss (405 Light On) values compared to the non-viricidal data (405 Light Off) shown in Table 5. These data are displayed in units of 1/h.

The data in Figure 7 display the non-viricidal and total loss k values for each surface in both dew-point conditions. The total loss k values represent the ‘405 Light On’ trendlines and the non-viricidal k values represent the ‘405 Light Off’ trendlines in Figure 2, Figure 3, Figure 4, Figure 5 and Figure 6. The high-dew-point conditions yielded higher total loss and non-viricidal k values for all surface types.

The ceramic surface displayed the greatest variation between the non-viricidal effects of dew points with k values of 0.855 and 0.122 1/h for the high and low dew points, respectively. The variations of the total loss for the high and low dew points were 1.09 and 0.341 1/h, respectively. The effects of the dew points were significant for the total and non-viricidal losses on the ceramic surface.

The PTFE disks displayed the highest total loss k values (1.38 and 0.523 1/h) for the high and low dew points, respectively. The non-viricidal losses were low compared to the total losses on the PTFE surface.

The stainless-steel disks displayed a moderate variation between the non-viricidal and total loss k values, 0.935 and 0.461 1/h, respectively, for the high-dew-point conditions. Interestingly, the surface displayed the lowest variation between the total losses and non-viricidal k values in the low-dew-point environment, indicating a minimal effect of BL_405_ in these conditions.

Statistical testing was conducted on these results and the values are shown in Figures S3 and S4 in the Supplementary Materials. These tests revealed that time exposure, dew point, and blue light were highly significant. Several interactions between these factors were also significant, including the effect of dew point over time, surface type and blue-light inactivation, blue-light dose, and the effect of the dew point on blue-light inactivation. For more detailed information on the statistical outputs, see the Supplementary Materials.

The results of these studies suggest that viral loss on ceramic, PTFE, and stainless-steel surfaces is a result of BL_405_ inactivation and viral degradation due to the long exposure times required to reach the desired dose. These novel results highlight that surface type and environmental conditions, such as how the dew point greatly affects the efficacy of BL_405_ inactivation technologies. The mechanisms of the variations observed by surface type and dew point are addressed below.

## 4. Discussion

The results of this work revealed many important findings. These studies assist in explaining the mechanisms of BL_405_ viral inactivation and provide evidence that its efficacy is impacted by environmental conditions (dew point), prolonged time exposure, and surface type. The viricidal k values reported for this work were significantly lower than the viricidal k values for the MS2 bacteriophage with UV_254_ [19,31]; this was due to the different inactivation mechanisms associated with these wavelengths.

The viricidal k values determined in this work (Table 4) fell between k values that were tabulated from the literature [13,14,24,32], ranging from 0.0013–0.1762 cm^2^/J. These k values ranged two orders of magnitude as they were calculated for five different viruses in many different media conditions. Rathnasinghe et al. [14] studied 405 inactivation of SARS-CoV-2, Influenza A, and encephalomyocarditis virus in liquid PBS. Tomb et al. [13] experimented with several media, including liquid PBS, artificial saliva, plasma, fetal bovine serum, and artificial feces. Lau et al. [24] and Vatter et al. [32] studied bovine coronavirus and Phi-6 bacteriophage, respectively, in liquid PBS. It should be noted that these k values were calculated for the experiments conducted in water; therefore, direct comparisons could not be made to the surface inactivation k values reported in these studies.

This work revealed many novel findings related to viral inactivation mechanisms and the impact of surface characteristics and the dew point. It was discovered that all the surface types achieved greater inactivation at the high dew points, compared to the low dew point. Additionally, the PTFE surface achieved the highest level of inactivation, whereas the ceramic surface achieved the lowest. This was thought to be due to the reflectivity, porosity, zeta potential, and contact angles of these surfaces. The effects of surface characteristics also impacted viral degradation over time; these findings are discussed in detail below.

Additionally, the surfaces were not allowed an inoculum drying period prior to experimentation. The literature [33,34,35] reports several studies on the effects of inoculum drying time before photoinactivation. SARS-CoV-2 studies suggest that the inactivation rate of SARS-CoV-2 is unaffected by drying prior to UV treatment [33,34]. This was likely due to the short drying times and UV dosing times utilized for these experiments. A study on SARS-CoV-2 and MS2 Bacteriophage using pulsed UV for inactivation reported varying results. The SARS-CoV-2 achieved higher inactivation rates with a wet inoculum, yet no effect of drying time was observed for the MS2 results [35]. These studies indicate that the inoculum drying time deserves consideration as it may influence the photoinactivation results.

### 4.1. Viral Inactivation Mechanisms

This work shows that BL_405_ has virucidal properties on ceramic, PTFE, and stainless-steel surfaces. Although the research in this field is sparse, some researchers [13,24,25,32] believe the dominant viral inactivation mechanism by 405 nm light exposure is by oxidative stress on the viral proteins. This damage can occur on both non-enveloped and enveloped viruses via damage to the viral capsid or viral envelope, respectively. It is thought that photosensitizers, or materials with porphyrin-like structures, must be present in the immediate surrounding environment and absorb the 405 nm wavelength. This interaction creates reactive oxygen species (ROS) that create stress on the proteins that comprise the viral capsid, or the viral envelope, leading to viral inactivation [13,24,25,32]. Viral envelopes consist of a lipid bilayer that encapsulates the viral capsid. The exterior of the bilayer is coated with a layer of glycoproteins [36]. Therefore, the extrapolation of the ROS mechanism from a non-enveloped virus to an enveloped virus is valid. These mechanisms may not transfer to viruses without protein coats, such as viroids [37] and Hepatitis D [38]. Further studies on viruses without protein coats are recommended.

Tomb et al. [13] conducted a comprehensive study on BL_405_ to observed the inactivation of feline calicivirus (non-enveloped), exploring many possible mechanisms for viral degradation during experimentation. The first experiments suspended the virus in a phosphate-buffered solution and exposed the samples to doses of 0, 561, 1683, and 2804 J/cm^2^. The highest 405 nm dose (2804 J/cm^2^) achieved a 3.9 log inactivation of feline calicivirus, with a calculated k value of 0.0013 cm^2^/J [13].

Additional experiments were conducted with the virus suspended in various organically rich media, such as artificial saliva, blood plasma, and artificial feces. The experiments using a virus suspended in an artificial saliva solution achieved a 2.25 log reduction at 280 J/cm^2^, with a corresponding k value of 0.0129 cm^2^/J. When suspended in a blood plasma solution, the virus achieved a 4.8 log inactivation at 561 J/cm^2^ (k value = 0.0087 cm^2^/J). Finally, when suspended in an artificial feces solution, the feline calicivirus achieved a 4.5 log inactivation at 1402 J/cm^2^, with a k value of 0.0032 cm^2^/J [13].

From these results, the inactivation of the feline calicivirus is greater when suspended in organically rich mediums. The highest inactivation level was observed using the artificial saliva solution. Tomb et al. [13] reported that the organic material in these solutions may have been sensitive to 405 nm, absorbing this wavelength and producing reactive oxidative species (ROS). The creation of ROS caused oxidative stress on the viral proteins, rendering it inactivated [13].

Another paper [32] studied the viricidal effects of BL_405_ on the Phi-6 bacteriophage, a surrogate for SARS-CoV-2. The Phi-6 bacteriophage is an enveloped dsRNA virus and is selected as a viral surrogate as the blue-light inactivation mechanisms of bacteriophages and mammalian viruses are thought to be similar. The goal of this study was to intentionally exclude photosensitizers to reveal the effects of BL_405_ without enhancement from extraneous material in the solution. The Phi-6 bacteriophage was suspended in phosphate-buffered saline (PBS) and a saline magnesium gelatin buffer. After exposure to a blue-light dose of 1300 J/cm^2^, a 3 log inactivation of the virus was observed in both solutions. When suspended in PBS, the calculated k value for the Phi-6 inactivation was 0.0026 cm^2^/J. The author suggested that the mechanism responsible for the inactivation of this bacteriophage was the material from the host cell cultures. The host cell for the Phi-6 bacteriophage is *Pseudomonas syringae*, whereas the host cell for the MS2 bacteriophage is *E. coli.* Both bacterial species were grown in tryptic soy broth. These cells had photosensitizers similar to porphyrins and may have been responsible for the inactivation observed [32].

The results of Vatter et al. [32] and Tomb et al. [13] relate to the findings of this research. *E. coli*, a bacterium and host cell for the MS2 bacteriophage, may have porphyrins that react to blue-light wavelengths. Portions of the *E. coli* cells and culture media were likely present in the MS2 solution applied to the surfaces. Figure S5 in the Supplementary Materials displays the correlation between the total organic carbon (TOC) of MS2 growth media at varying concentrations and its corresponding absorbance at 405 nm. The TOC concentration in the solution used to inoculate the surfaces was 130 mg/L, corresponding to 0.0231 cm^−1^ at a 405 nm absorbance. It is likely that portions of the *E. coli* cells and organic material from the MS2 broth solution acted as photosensitizers upon the interaction with 405 nm wavelengths.

Another mechanism proposed by Tomb et al. [13] suggests that the inactivation of the feline calicivirus in the PBS solution may have been due to inactivation from extraneous wavelengths outside of the 405 nm band. The typical spectral distribution of wavelengths for commercially purchased blue-light collimate beams commonly seen in the literature [13,14] falls between 390–430 nm, with the peak irradiance output at 405 nm. It has been reported that 390 nm causes oxidative stress to the viral capsid and other proteins [39]. Wavelengths between 420–430 nm were found to cause the inactivation of the murine leukemia virus (enveloped), due to damage to the virion-associated reverse-transcription complex [40]. Therefore, over the long exposure times associated with these experiments, these wavelengths may have caused the inactivation observed [13]. Additionally, a 2021 paper tested the effects of a 405 nm viral inactivation on SARS-CoV-2 (enveloped) and Influenza A Virus (enveloped) without the use of any photosensitizers and confirmed that inactivation by UVA (~390 nm) and 420–430 nm contributed to viral losses [14].

This provides a further insight into an additional inactivation mechanism responsible for the degradation of the MS2 bacteriophage on our surfaces. As shown in the Supplementary Materials in Figure S6, 8.3% of the energy emitted by the Thorlabs collimated LED fall in the UVA range. A total of 91.5% of the irradiance fell within the blue-light range, with 10.3% falling between 420–430 nm. Regardless of the presence of photosensitizers in the inoculum solution, these wavelengths may have contributed to all or part of the inactivation of the MS2 bacteriophage observed on ceramic, PTFE, and stainless-steel surfaces.

### 4.2. Surface Characteristics

The viricidal effects of BL_405_ and non-viricidal viral degradation also varied by surface type. Several surface characteristics were explored to explain the causal mechanisms behind the observed variations.

The MS2 inactivation was significantly higher on the PTFE surface for both the high and low dew points, compared to the ceramic and stainless-steel surfaces. The reflectivity of PTFE was studied for wavelengths ranging from 250–500 nm and was reported to be approximately 97% [41]. For comparison, the visible-light reflectivity for stainless steel was approximately 50% [42]. These high-reflectivity values allowed the wavelength to continuously reflect as it contacted the surface, and therefore could move across the surface and into pore spaces more easily. This phenomenon has been studied in the UV_254_ literature [2,19,43,44] and is reported to increase the contact between UV light and viral particles, dramatically increasing inactivation.

The ceramic surface achieved the lowest viricidal effect of BL_405_ for both the high- and low-dew-point experiments. This was likely due to the surface porosity of this material. Figure S7 in the Supplementary Materials display the porosity values of each surface. The ceramic surface is the most porous of the three surfaces, with a surface porosity of 17.3%, compared to 1.6% and 2.9% for PTFE and stainless steel, respectively. The literature [45] concerning UV_254_ studies indicates that surfaces with a higher surface porosity yield lower the photoinactivation levels better than non-porous surfaces. This phenomenon has been named the ‘Canyon wall effect’ [45] due to the relative size of the virus particle, approximately 27 nm [46], compared to the larger surface pores, which allow the viral particles to penetrate deeper into the surface. The pore spaces shield viral particles from UV wavelengths, therefore reducing the level of photoinactivation that can occur on the surface [19,45]. As shown in Table 4, this phenomenon is pertinent to the BL_405_ data as well.

Additionally, as shown in Figure 2, Figure 3 and Figure 7, non-viricidal viral degradation over time is higher on the ceramic surface compared to the PTFE and stainless steel. The literature suggests that non-viricidal viral degradation over time increases on porous surfaces [47,48]. It has been found that coronaviruses are less stable on porous surfaces than non-porous surfaces over time. This is thought to be due to adsorption onto the surface, electrostatic interactions, and intermolecular effects, such as van der Waals forces [47]. Figure S8 in the Supplementary Materials display a correlation between the viral degradation and porosity, such that as the porosity increases, the viral degradation increases.

Figure 7 shows that these findings are exaggerated in high-dew-point environments, specifically for the ceramic surface. As shown by this work, viral degradation increases as the temperature increases. This phenomenon may be amplified by porous surfaces. As the temperature increases, the viscosity of the virus inoculum decreases. Therefore, it follows that higher-temperature environments promote the further penetration of the inoculum into the porous surface. This likely heightened the viral degradation due to the forces mentioned above, as the virus had a greater surface area for interactions with the surface. Additionally, van der Waals forces have been reported to become stronger due to increasing long-range molecular interactions as the temperature increases [49], resulting is a stronger viral attachment to the surface.

The contact angles of the ceramic, PTFE, and stainless-steel samples are shown in Figures S9–S11 in the Supplementary Materials. The contact angle is a measure of hydrophilicity/hydrophobicity. Surfaces with lower contact angles are more hydrophilic, whereas surfaces with higher contact angles are more hydrophobic [50]. Figure S9 displays the correlation for high-dew-point conditions between the viricidal k value and contact angle of each surface, such that as the contact angle increases, the viricidal effectiveness increases. This can be explained by the literature [51] as surfaces with lower contact angles are conducive to an increased penetration of the inoculum and viral attachment, whereas surfaces with higher contact angles may retain the inoculum as a droplet on the surface. The surfaces with the higher contact angles retained a greater portion of the virus and inoculum on the top of the surface, therefore allowing the increased exposure of the inoculum and virus to the BL_405_ wavelengths, creating more ROS, thus leading to higher levels of inactivation. This is consistent with the high-dew-point results presented in this paper, as the stainless-steel and PTFE surfaces had higher contact angles and achieved higher viricidal inactivation values.

Figure S10 in the Supplementary Materials displays viral degradation as a function of the contact angle. This figure shows that, as the contact angle increases, the viral degradation decreases. The literature reveals that the contact angle is affected by the porosity of the material [52,53,54]. Figure S11 in the Supplementary Materials shows a strong correlation between the contact angle and porosity (R^2^ = 0.996), such that as the contact angle increases, the porosity decreases. Therefore, after understanding this relationship, Figure S11 reinforces the findings of Figure S8 and the literature stating that more porous surfaces yield lower viral stability results over time.

The zeta-potential data are shown in Figures S12 and S13 in the Supplementary Materials. Figure S12 displays a strong correlation (R^2^ = 0.999) between the viricidal k values and zeta potential, such that as the zeta potential increases, viricidal k decreases. This follows as the literature [54] suggests that surfaces with more positive zeta potentials will have higher negative viral depositions on the surface. The MS2 bacteriophage is a negatively charged virus [55]; therefore, surfaces with more negative zeta potentials, such as PTFE, will have a weaker attraction of negative viral particles to the surface. Consequently, the virus and inoculum are more exposed to BL_405_ wavelengths. Increased exposure to BL_405_ increases the production of ROS, thus increasing the inactivation of the MS2 bacteriophage on the surface.

Finally, Figure S13 displays the correlation between the zeta potential and the non-viricidal k value. The figure shows that as the surface charge becomes less negative, the non-viricidal k value increases. This follows as the negatively charged MS2 virus is more likely to adsorb/bond with a surface with a less negative charge. Increased adsorption on the surface yields lower recovery values, thus increasing the non-viricidal k value.

### 4.3. Increased Inactivation in High-Dew-Point Conditions

Another significant finding of this research revealed the importance of the dew point regarding BL_405_ inactivation efficacy. This work demonstrates that BL_405_ inactivation technologies are more effective in high-dew-point environments. This phenomenon was observed with ceramic, PTFE, and stainless-steel surfaces. This finding supports the proposed dominant inactivation mechanism of BL_405_ and is confirmed by atmospheric studies in the literature that demonstrate that reactive oxidant species increase as the moisture content increases [56,57]. The research conducted by Lespade et al. [58] demonstrated the importance of water molecules in the oxidation of organic molecules by a superoxide anion. This work demonstrated that the first layer of surrounding water molecules was essential for reactivity, as it decreased the energy of the excited states of the hydrated superoxide anion and facilitated the reaction with organic molecules [58]. At high relative humidities, there was an increase in the soluble oxygen available for the reaction with blue light.

Three studies in other disciplines [56,57,59] support the theory that as the relative humidity increases, the creation of reactive oxygen species increases. This research revealed increased virucidal k values in high-dew-point environments. These findings are consistent with the literature [13,24,25,32] that suggests that ROS is the dominant mechanism responsible for the BL_405_ inactivation of viruses.

### 4.4. Viral Degradation over Time: Temperature and Relative Humidity

This work highlighted the importance of conducting controls, or time exposure tests, for BL_405_ experiments. These experiments required long exposure times (in the order of hours), due to the high dose needed and the limited irradiance that could be provided by BL_405_ sources. Previous studies [14,24,32] reported that viral degradation over time was not a significant source of viral loss, although our work suggested otherwise. The studies mentioned were conducted in liquid media, which likely contributed to the disparity in the results of the control experiments on surfaces. Additionally, the irradiance values used by Lau et al. [24] (144.4 mW/cm^2^) and Vatter et al. [32] (78.6 mW/cm^2^) were significantly higher than the 13 mW/cm^2^ BL_405_ irradiance output used for our studies. The high-irradiance values decreased the required exposure time for these experiments, likely limiting the viral degradation.

This paper demonstrated that significant viral degradation over time in the absence of BL_405_ occurred on all surfaces and was exacerbated by the high-dew-point conditions. The effects of viral degradation as a function of temperature and dew point are reported in the literature [22,60,61,62]. In this research, high-dew-point experiments were conducted at 28 °C. A 2018 study [62] quantified the effects of relative humidity and temperature on Phi-6 bacteriophage recovery and found similar results to those reported in this paper. The highest recovery was found with relative humidities below 60% or above 80%. When the relative humidity was set at 75% and the temperature increased from 19 °C to 25° C, the infectivity of Phi-6 decreased by two orders of magnitude [62].

Another study [22] using SARS-CoV-2 supported the results found in this paper. This study tested varying relative humidity and temperature conditions on polypropylene plastic surfaces. The results of this study found that viral decay increased as the temperature increased, regardless of the relative humidity. The relative humidity had a lesser effect on viral decay compared to temperature. The effect of relative humidity appeared to be U-shaped, in that viral decay was the highest at a 65% relative humidity and lower at 40% and 85% relative humidity levels [22]. The author suggested that the low and high relative humidity conditions hindered the ionic equilibrium, which directly impacted the evaporation rate of the droplet [22].

Temperature effects have also been observed with the MS2 bacteriophage. A 2012 study [61] looked at the structural integrity of MS2 after various inactivation techniques. These experiments were conducted in a PCR thermocycler for 2 min. An 8.5 log inactivation of the MS2 bacteriophage was observed and was found to exist due to structural damage to the proteins responsible for binding to the host cell. The virus remained infectious outside of the host cell but could not replicate to produce progeny [61].

Additionally, viral samples sent to the partnering laboratory for this work, GAPLAB, in Ontario, Canada, must comply with their QA/QC requirements for temperature. The samples must arrive at the laboratory between 2–10 °C; samples outside that range cannot be analyzed due to sample non-conformance. This requirement reiterates that the MS2 bacteriophage is sensitive to increased temperatures. As the temperature increases, MS2 stability decreases [29,63].

The data gathered from the work of Morris et al. [22], Wigginton et al. [61], Prussin et al. [62], and EPA [29,63] strongly suggest that the higher degradation of MS2 in the high-dew-point experiments is likely due to the higher temperature, 28 °C as compared to 10 °C in the low-dew-point conditions. The viral proteins likely degraded due to the high temperature and long exposure times of these experiments, rendering a portion of the MS2 bacteriophage unable to infect the host *E. coli*. The low-dew-point environments may have caused the desiccation of the MS2 virus [64]; yet, these effects were highly variable.

Finally, this research contributed the necessary data to the photoactivation industry regarding the effects of surface characteristics and dew points on BL_405_ inactivation efficacy. When properly designed, BL_405_ technologies may provide an extra layer of protection against viral pathogens. However, the long exposure times required for adequate viral inactivation (2–3 log) may suggest that these technologies cannot keep up with the viral shedding of an infected individual. The approximate viral shedding rate of a person infected with SARS-CoV-2 is 1800 pathogens/h [65]. Depending on the surface type and environmental conditions, it may be beneficial to utilize an additional inactivation technology concurrently with BL_405_ for the optimum protection of human health.

The following citations can be found in the Supplementary Materials [14,19,24,25,26,66,67,68,69,70,71,72,73,74,75].

## 5. Conclusions

This work uncovered many important findings regarding the efficacy of BL_405_ surface inactivation. The major findings are listed below:

- This research provides sufficient evidence that BL_405_ has a viricidal effect on the MS2 bacteriophage.
- The high-dew-point conditions yield higher levels of inactivation of the MS2 bacteriophage on all the surfaces tested.
- Conducting time exposure controls, represented by the ‘405 Light Off’ data, is prudent for determining the true viricidal effect of BL_405_. Due to the long exposure times required, viral degradation occurs on the surface without exposure to BL_405_. These effects increase in the high-dew-point conditions.
- The highest level of inactivation due to BL_405_ (3.9 log) is observed on the PTFE surface, in the high-dew-point conditions.
- The characteristics (zeta potential, contact angle, and porosity) of each surface impact BL_405_ inactivation efficacy and viral degradation over time.
  ○ As the zeta potential increased, the viricidal effects of BL_405_ decreased, and viral degradation increased in the high-dew-point conditions.
  ○ As the contact angle increased, the viricidal effects of BL_405_ increased, and viral degradation decreased in the high-dew-point conditions.
  ○ As the porosity increased, viral degradation increased in the high-dew-point conditions.

## Figures and Tables

**Table 1:** Surfaces utilized for experimentation.

Surface Type	Manufacturer/Description
Ceramic	Ceramic Solutions (Conroe, TX, USA): 99.8% Alumina Discs
PTFE	Thorlabs (Newton, NJ, USA): Fabricated from sintered, crystalline, fused, and skived virgin PTFE
Stainless steel	MetalsDepot (Winchester, KY, USA): 304 Stainless with Mirror Finish (#8)

**Table 2:** Temperature and relative humidity conditions in controlled chamber.

Dew Point (°C)	Temperature (°C)	Relative Humidity (%)
−4	12	32
18	28	55

**Table 3:** BL_405_ doses and exposure times.

**Doses (J/cm^2^**)	**0**	**50**	**100**	**200**
Exposure time (hour, min)	0	1 h 4 min	2 h 8 min	4 h 16 min

**Table 5:** Non-viricidal k values for each surface by dew point.

Surface	High-Dew-Point k (1/h)	Low-Dew-Point k (1/h)
Ceramic	0.8550	0.1221
PTFE	0.4109	No Data
Stainless steel	0.4492	0.2076

## Data Availability

For access to the file containing all raw data collected during experimentation, please visit https://dx.doi.org/10.34051/d/2023.2.

## Supplementary Materials

The following supporting information can be downloaded at: https://www.mdpi.com/article/10.3390/microorganisms11112638/s1. Figure S1: displays the BL_405_ dose response curve for six viral species. BL_405_ dose (0–260 J/cm^2^) is shown on the x-axis and log inactivation is displayed on the y axis; Figure S2: displays the log loss of MS2 bacteriophage as a function of time on stainless steel disks in a low dew point environment. Time in hours is shown on the x axis and log loss of MS2 is shown on the y axis. Two trendlines are displayed, indicating the log loss of MS2 as a function of time with and without exposure to BL_405_ irradiance. The shaded region between the two trendlines represents the viricidal effect of BL_405_ on stainless steel disks in low dew point environments. The microbial detection limit for these experiments is shown as a line on the top of the figure; Figure S3: displays the results of a full factorial analysis conducted in JMP; Figure S4: displays the results of the statistical analysis performed in JMP. Statistical significance was determined by p values greater than 0.05; Figure S5: displays the concentration of total organic carbon (TOC) in mg/L versus the BL_405_ absorbance (cm^−1^). The TOC concentration is on the x axis and the BL_405_ absorbance is on the y axis; Figure S6: displays the irradiance distribution of the Thorlab 405 nm collimated beam. The irradiance measurements were recorded with an ILT960 Spectroradiometer; Figure S7: displays the surface porosity (measured with SEM) of ceramic, PTFE and stainless steel; Figure S8: displays the surface porosity of ceramic, PTFE, and stainless steel as a function of their non-viricidal k values in high dew point environments; Figure S9: displays the viricidal k value as a function of the contact angle of ceramic, stainless steel and PTFE; Figure S10: displays the correlation between the contact angle of ceramic, stainless steel, and PTFE and non- viricidal k values in high dew point environments; Figure S11: displays contact angle versus porosity for aluminum, ceramic, Formica laminate, PTFE, and stainless steel. Contact angle (°) is shown on the x axis. Percent surface porosity is shown on the y axis; Figure S12: displays the zeta potential (mV) of PTFE, stainless steel, and ceramic versus the viricidal k values in the high dew point environments; Figure S13: displays the zeta potential (mV) for PTFE, stainless steel, and ceramic as a function of the non-viricidal k values in the high dew point conditions; Table S1: displays the characteristics of the viruses shown in Figure S1. The characteristics of MS2 bacteriophage were included in this table for comparison.
