# Supplementary material for: Impacts of Surface Characteristics and Dew Point on the Blue-Light (BL405) Inactivation of Viruses
Source: Microorganisms. 2023 Oct 26;11(11):2638. doi: 10.3390/microorganisms11112638 (PMC10673487; doi:10.3390/microorganisms11112638)
Supplement: Supplementary file 1 [file microorganisms-11-02638-s001.zip › microorganisms-2650038-supplementary.pdf]

Supplemental Information for *Impacts of Surface Characteristics and Dew Point on Blue Light (BL_{405}) Inactivation of Virus*

Castine Bernardy, James Malley

University of New Hampshire

1. Additional Information- Methods and Materials

1.1 Humidifier and Dehumidifier

The humidifier utilized for this work was manufactured by Sunbeam, model SCM3502. When the weather conditions called for additional assistance to maintain the high humidity, an AquaOasis Cool Mist Humidifier (model AO-101) was placed in close proximity to the collimated beam. Additionally, the dehumidifier to maintain the low dew point was manufactured by BUBLUE, model OL20D- D029A.

2. Figures:

2.1 BL_{405} Dose

The BL_{405} doses selected were based off previous viral BL_{405} inactivation studies in the literature [14, 24-26]. Figure S1 displays the blue light dose response curve of 6 viral species.

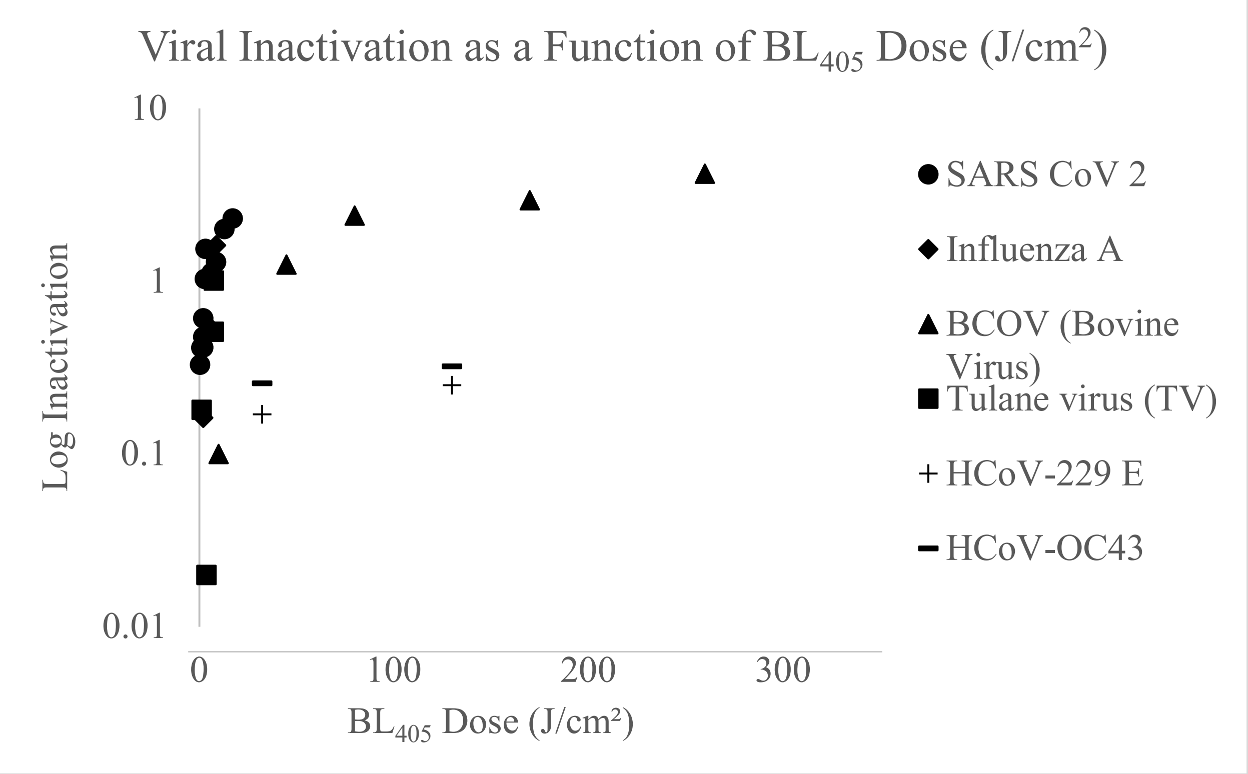

Figure S1. displays the BL_{405} dose response curve for six viral species. BL_{405} dose (0-260 J/cm^2) is shown on the x-axis and log inactivation is displayed on the y axis.

Figure S1 displays the BL_{405} dose response of SARS-CoV-2, Influenza A, Bovine Virus, Tulane Virus, HCoV-229E, and HCoV-OC43. These data were collected from the available literature [14, 24-26] on viral inactivation with BL_{405}. These data were used to inform our selection of BL_{405} doses for MS2 bacteriophage inactivation. The characteristics of each viral species shown in Table S1 below.

Table S1. displays the characteristics of the viruses shown in Figure S1. The characteristics of MS2 bacteriophage were included in this table for comparison.

**Virus**	**RNA/DNA**	**ss/ds**	**Size (nm)**	**Enveloped?**
Human alpha coronavirus HCoV-229E	RNA	Ss	80-120	Enveloped
Human beta coronavirus HCoV-OC43	RNA	Ss	80-120	Enveloped
SARS-CoV-2	RNA	Ss	50-140	Enveloped
Bovine Coronavirus	RNA	Ss	65-210	Enveloped
Influenza A	RNA	Ss	80-120	Enveloped
Tulane virus	RNA	Ss	40	Non-enveloped
Feline calicivirus	RNA	Ss	35-38	Non-enveloped
MS2 Bacteriophage	RNA	Ss	27	Non-enveloped

The characteristics of each virus displayed in Figure S1 were tabulated from the literature [66-75] and are displayed in Table S1. These characteristics were compared to the characteristics of MS2 bacteriophage to determine the most structurally similar virus. Tulane virus and Feline calicivirus are the most similar to MS2 bacteriophage, as they are ssRNA viruses of similar size and non-enveloped. The doses used in the study using Tulane virus ranged from approximately 1-8 J/cm^2, which did not achieve higher than 1 log inactivation. One dose was tested for Feline calicivirus, 2800 J/cm^2 and achieved 4 log inactivation (not pictured in Figure S1). The doses used for Tulane virus did not achieve adequate disinfection (3 log), therefore, the doses used in these experiments were increased accordingly, ranging from 0-200 J/cm^2.

2.2 Stainless Steel Dose Response Curve

The dose response curve for stainless steel in low dew point conditions is shown below in Figure S2.

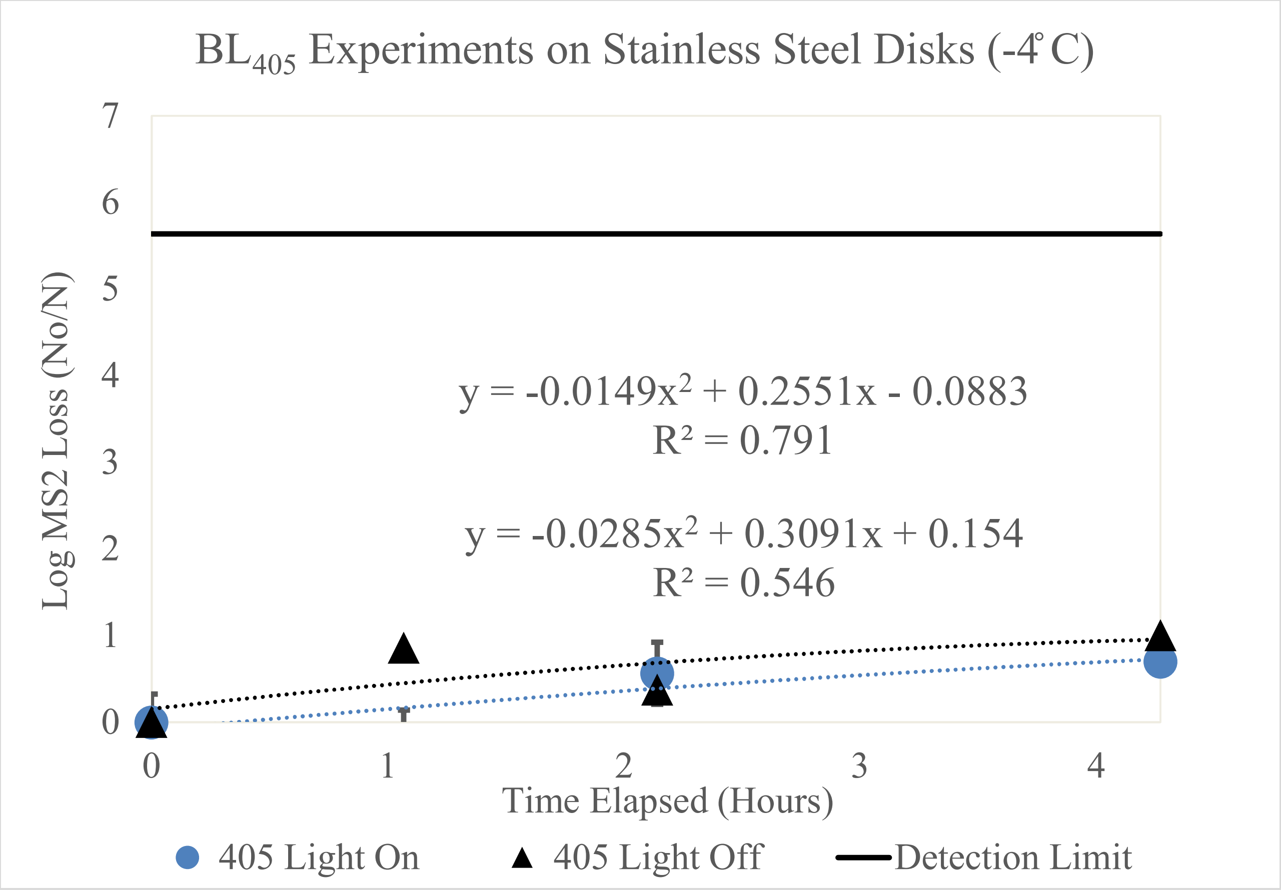

Figure S2. displays the log loss of MS2 bacteriophage as a function of time on stainless steel disks in a low dew point environment. Time in hours is shown on the x axis and log loss of MS2 is shown on the y axis. Two trendlines are displayed, indicating the log loss of MS2 as a function of time with and without exposure to BL_{405} irradiance. The shaded region between the two trendlines represents the viricidal effect of BL_{405} on stainless steel disks in low dew point environments. The microbial detection limit for these experiments is shown as a line on the top of the figure.

The BL_{405} dose response curve for stainless steel disks is shown in Figure S2. This figure shows two trendlines, representing the loss of MS2 with (blue circle) and without (black triangle) exposure to BL_{405} irradiance. The BL_{405} irradiance corresponding to the '405 Light On' trendline was 13 mW/cm^2, therefore, are representative of BL_{405} doses of 0, 50, 100, and 200 J/cm^2. Three trials were conducted for each BL_{405} dose, Figure S2 displays the average of these trials and the standard deviation as error bars.

The blue trendline represents the experiments conducted with exposure to BL_{405} irradiance (total losses), whereas the black trendline represents the loss of MS2 accrued over time. These data points heavily intersected, suggesting that BL_{405} had no significant effect (NSE) on stainless steel disks in low dew point environments. The loss that occurred on the stainless steel in the low dew point environment was due to loss over time, although this loss was small.

Over 4.3 hours the total loss of MS2 on stainless steel was approximately 1 log in low dew point conditions.

2.3 Statistical Analysis

Statistical analysis in JMP 16.1 was conducted to determine if the effects of blue light, surface type, time exposure, and dew point significantly impacted the log loss of MS2 bacteriophage. A full factorial analysis was performed and is shown below in Figure S3.

Response Log Co/C

Actual by Predicted Plot

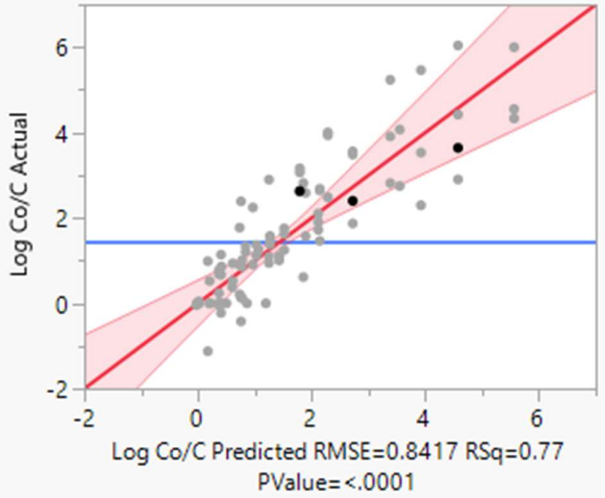

Effect Summary

Source	LogWorth	PValue	
Dew Point	8.725	0.00000	
Time (hrs)	8.193	0.00000	
Blue Light	4.572	0.00003	
Dew Point*Time (hrs)	3.016	0.00096	
Surface*Blue Light	1.858	0.01387	
Time (hrs)*Blue Light	1.655	0.02213	
Dew Point*Blue Light	1.630	0.02344	
Surface*Time (hrs)*Blue Light	0.670	0.21377	
Surface	0.555	0.27879	^
Dew Point*Time (hrs)*Blue Light	0.430	0.37166	
Surface*Dew Point	0.303	0.49786	
Surface*Time (hrs)	0.252	0.55976	^
Surface*Dew Point*Time (hrs)*Blue Light	0.203	0.62657	
Surface*Dew Point*Blue Light	0.156	0.69847	^
Surface*Dew Point*Time (hrs)	0.063	0.86565	^

Remove Add Edit ☐ FDR ('^' denotes effects with containing effects above them)

Lack Of Fit

Source	DF	Sum of Squares	Mean Square	F Ratio
Lack Of Fit	23	24.152714	1.05012	1.7993
Pure Error	63	36.767933	0.58362	Prob > F
Total Error	86	60.920647		0.0346*
				Max RSq
				0.8613

Figure S3. displays the results of a full factorial analysis conducted in JMP.

The results of a full factorial analysis are displayed in Figure S3. This analysis was conducted to determine the appropriate inputs to be included in the JMP output used for

determining the statistical significance of dew point, surface type, blue light, and time exposure. Per advisement of the UNH statistical consulting center, interactions or main effects with *p values* greater than 0.5 were removed from the model. Therefore, the last four effects in this model were excluded in the model used to determine significance, shown in Figure S4 below.

Response Log Co/C

Actual by Predicted Plot

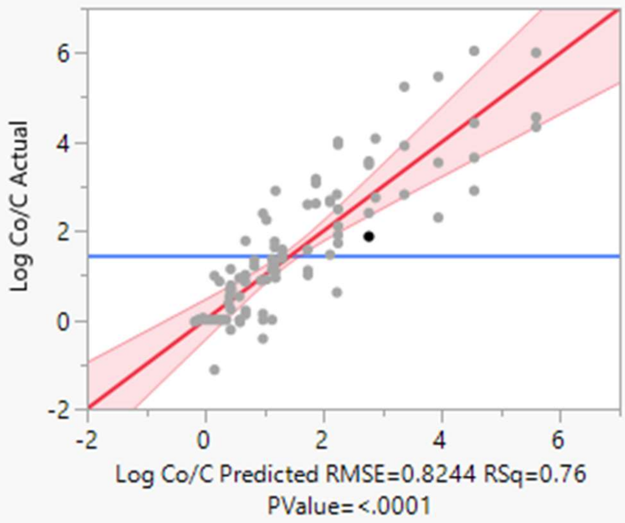

Effect Summary

Source	LogWorth		PValue
Time (hrs)	10.467		0.00000
Dew Point	10.209		0.00000
Blue Light	5.395		0.00000
Dew Point*Time (hrs)	4.091		0.00008
Surface*Blue Light	2.275		0.00530
Time (hrs)*Blue Light	2.260		0.00550
Dew Point*Blue Light	1.790		0.01621
Surface	0.987		0.10304 ^
Surface*Time (hrs)*Blue Light	0.749		0.17808
Dew Point*Time (hrs)*Blue Light	0.480		0.33135
Surface*Dew Point	0.272		0.53487

Remove Add Edit ☐ FDR ('^' denotes effects with containing effects above them)

Lack Of Fit

Source	DF	Sum of Squares	Mean Square	F Ratio
Lack Of Fit	31	27.111203	0.874555	1.4985
Pure Error	63	36.767933	0.583618	Prob > F
Total Error	94	63.879136		0.0873
				Max RSq
				0.8613

Figure S4. displays the results of the statistical analysis performed in JMP. Statistical significance was determined by p values greater than 0.05.

The statistical significance of dew point, surface type, blue light, and viral degradation were tested in the fit model platform in JMP. Statistical significance was determined for interactions with *p values* less than 0.05. The significant factors and interactions (listed in order of most to least significant) are as follows; time, dew point, blue light, dew point*time, surface*blue light, time*blue light, and dew point*blue light.

2.4 Total Organic Carbon (TOC)

Figure S5 below displays the total organic carbon versus absorbance at BL_{405}.

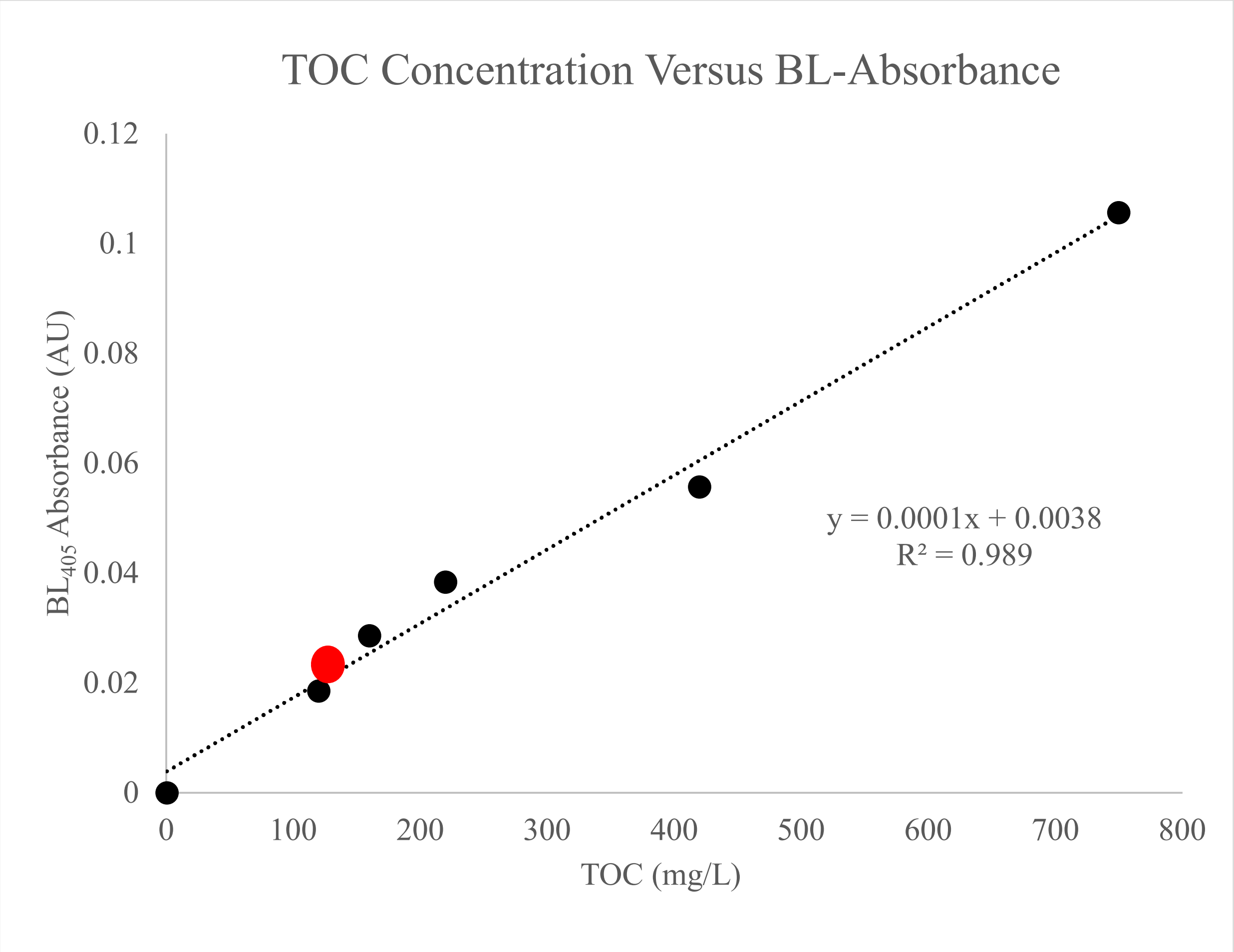

Figure S5. displays the concentration of total organic carbon (TOC) in mg/L versus the BL_{405} absorbance (cm^{-1}). The TOC concentration is on the x axis and the BL_{405} absorbance is on the y axis.

A strong correlation (R^2=0.989) between TOC concentration (mg/L) and BL_{405} absorbance (cm^{-1}) is displayed in Figure S5. As TOC concentration (mg/L) increases, blue light absorbance increases. The red marker on the figure displays the TOC concentration (130 mg/L) and corresponding BL_{405} absorbance (0.0231 cm^{-1}) of the inoculum applied to the surfaces. The

high levels of TOC in the inoculum likely contributed to the production of ROS on the surfaces leading to increased MS2 bacteriophage inactivation.

2.5 Irradiance Distribution Measurements

To measure the irradiance distribution of the Thorlabs collimated beam, an ILT 906 Spectroradiometer was utilized. The spectroradiometer is manufactured by International Light Technologies in Peabody, Massachusetts. The device can measure accurately measure irradiance between 180-850 nm.

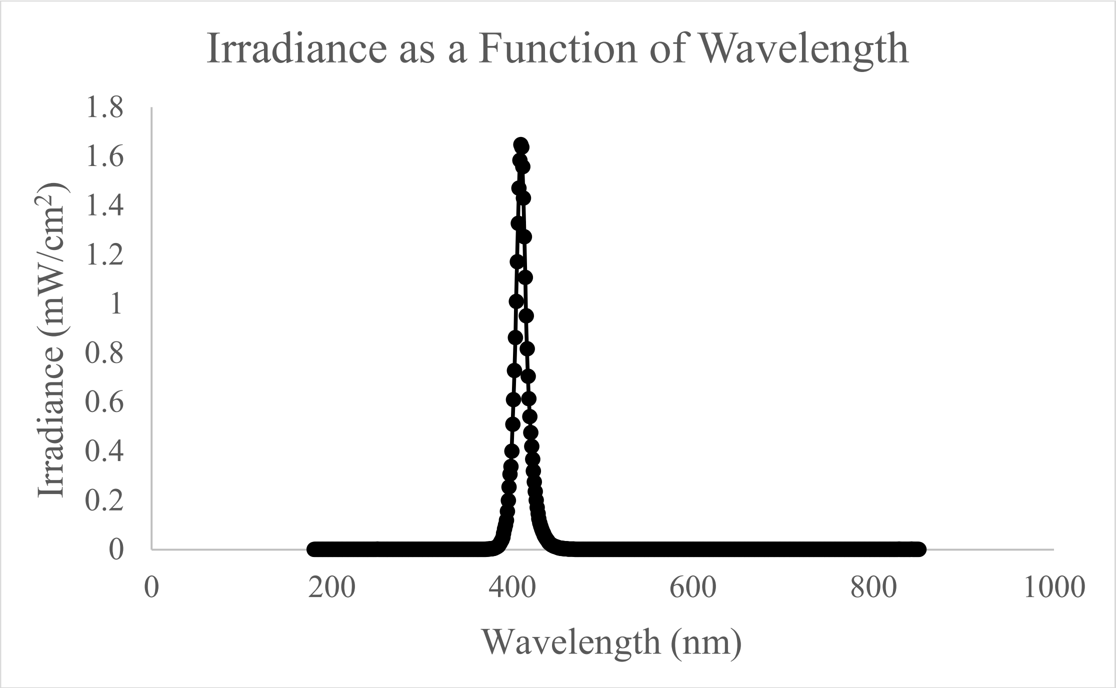

Figure S6. displays the irradiance distribution of the Thorlab 405 nm collimated beam. The irradiance measurements were recorded with an ILT960 Spectroradiometer.

Figure S6 displays the irradiance as a function of wavelength. These values were collected with an ILT 960 Spectroradiometer and measure the irradiance (mW/cm^2) from 180-850 nm. The peak irradiance output is 1.647 mW/cm^2 at 409 nm. 8.3 % of the irradiance fell inside of the UVA range (315-400 nm). 91.5 % of the irradiance fell in the blue light range, with 10.3 % between 420-430nm.

2.6 Surface Characteristics

The following figures display information on the characteristics of the surfaces used for experimentation. A detailed procedure of how these characteristics were quantified can be found in a previous publication, Bernardy et al. [19].

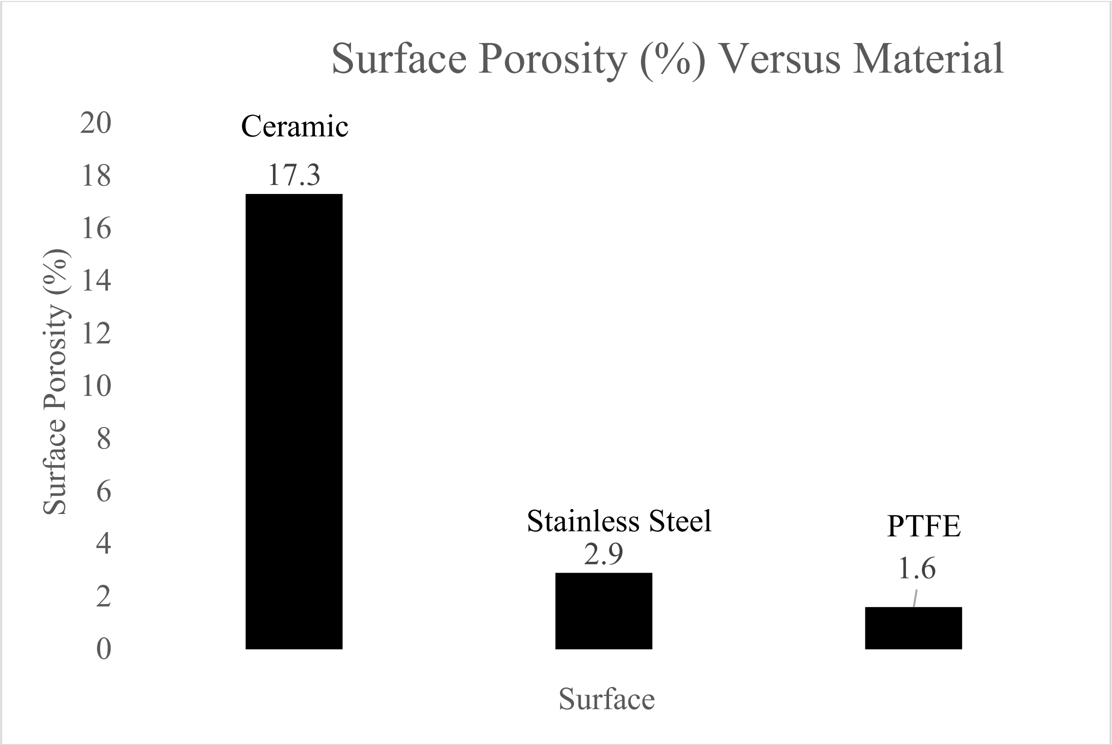

Figure S7. displays the surface porosity (measured with SEM) of ceramic, PTFE and stainless steel.

The surface porosity of each material is displayed in Figure S7. The ceramic material has the highest surface porosity (17.3%), followed by stainless steel (2.9%) and PTFE (1.6%).

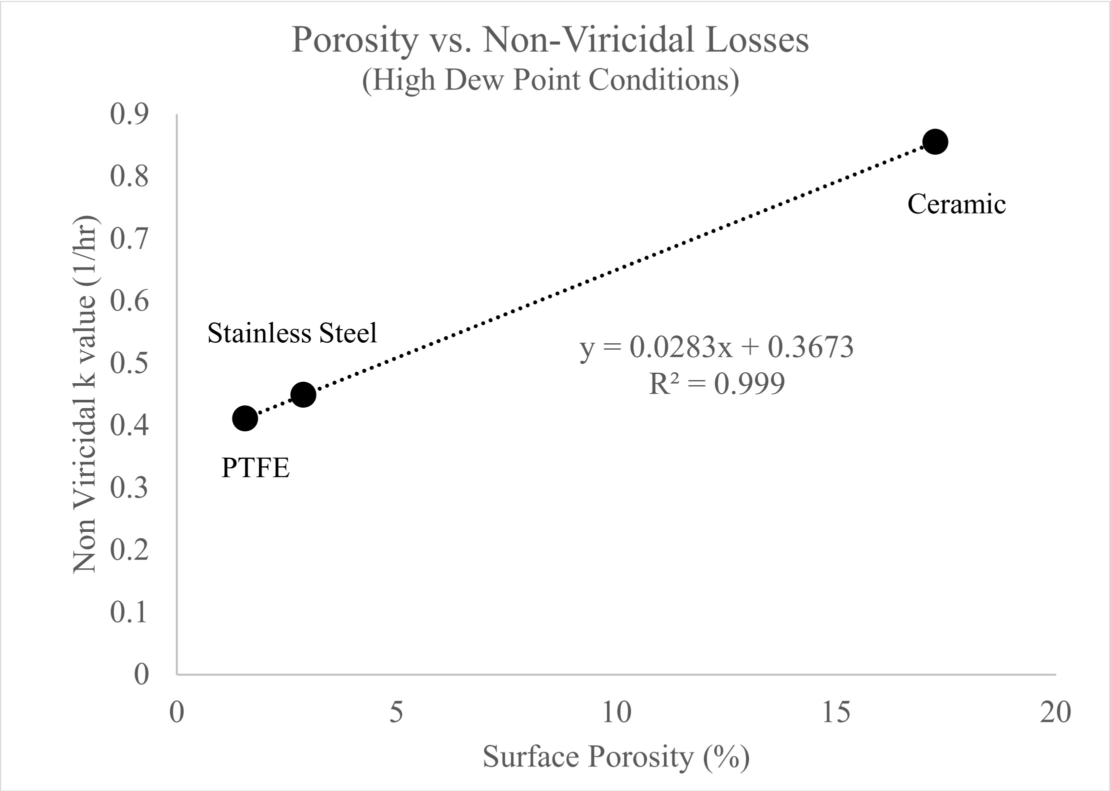

Figure S8. displays the surface porosity of ceramic, PTFE, and stainless steel as a function of their non-viricidal k values in high dew point environments.

The non-viricidal k of ceramic, PTFE, and stainless steel is shown as a function of surface porosity in Figure S8. This relationship was found for the non-viricidal k values in the high dew point environments. The figure displays a strong linear correlation (R^2=0.999), such that as surface porosity increases, the non-viricidal k increases. The highest non-viricidal k value and corresponding surface porosity was observed for ceramic. The PTFE had the lowest surface porosity, therefore had the lowest non-viricidal k value.

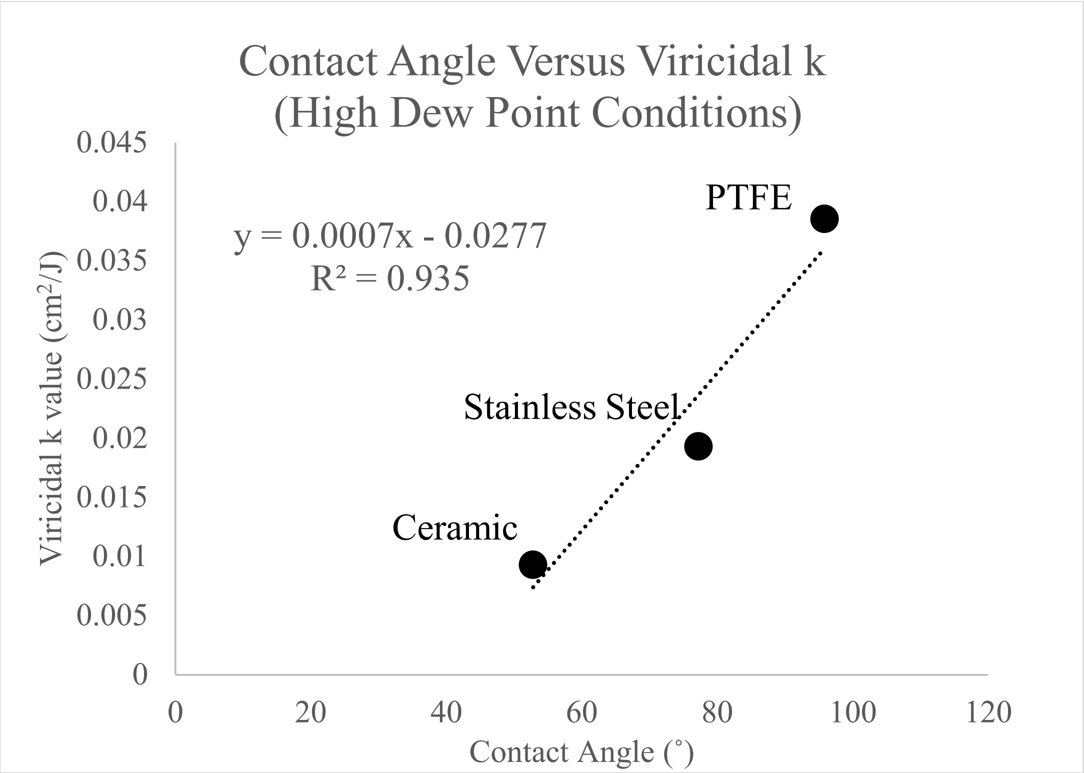

Figure S9. displays the viricidal k value as a function of the contact angle of ceramic, stainless steel and PTFE.

A strong linear correlation (R^2=0.935) between contact angle (˚) and viricidal k for ceramic, stainless steel and PTFE can be observed in Figure S9. This correlation is representative of the viricidal k in the high dew point conditions. As contact angle increases, the viricidal k for each surface increases. The PTFE surface had the highest contact angle and viricidal k value, whereas the ceramic surface had the lowest of these values.

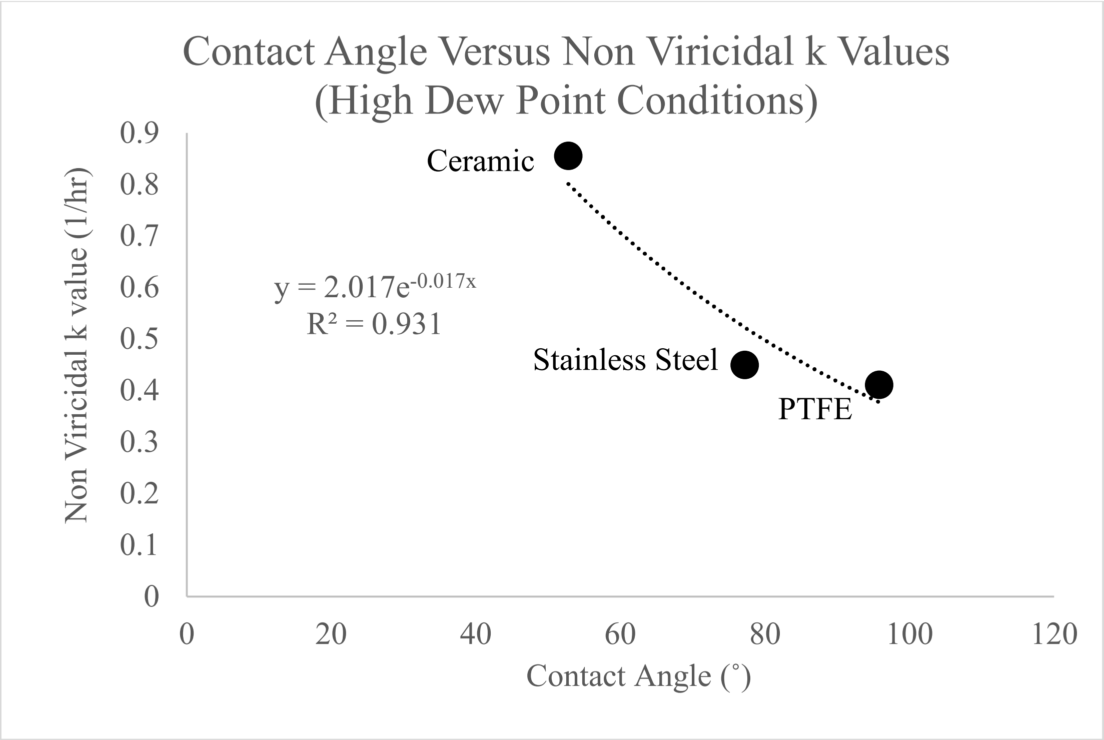

Figure S10. displays the correlation between the contact angle of ceramic, stainless steel, and PTFE and non- viricidal k values in high dew point environments.

A strong (R^2=0.931) non-linear relationship between contact angle and non-viricidal k can be observed in Figure S10. This relationship was found for the non-viricidal k values in the high dew point conditions. As contact angle increases, non-viricidal k decreases. The ceramic surface had the lowest contact angle, and the highest non-viricidal k value. Whereas the PTFE surface had the highest contact angle and the lowest non-viricidal k value in the high dew point conditions.

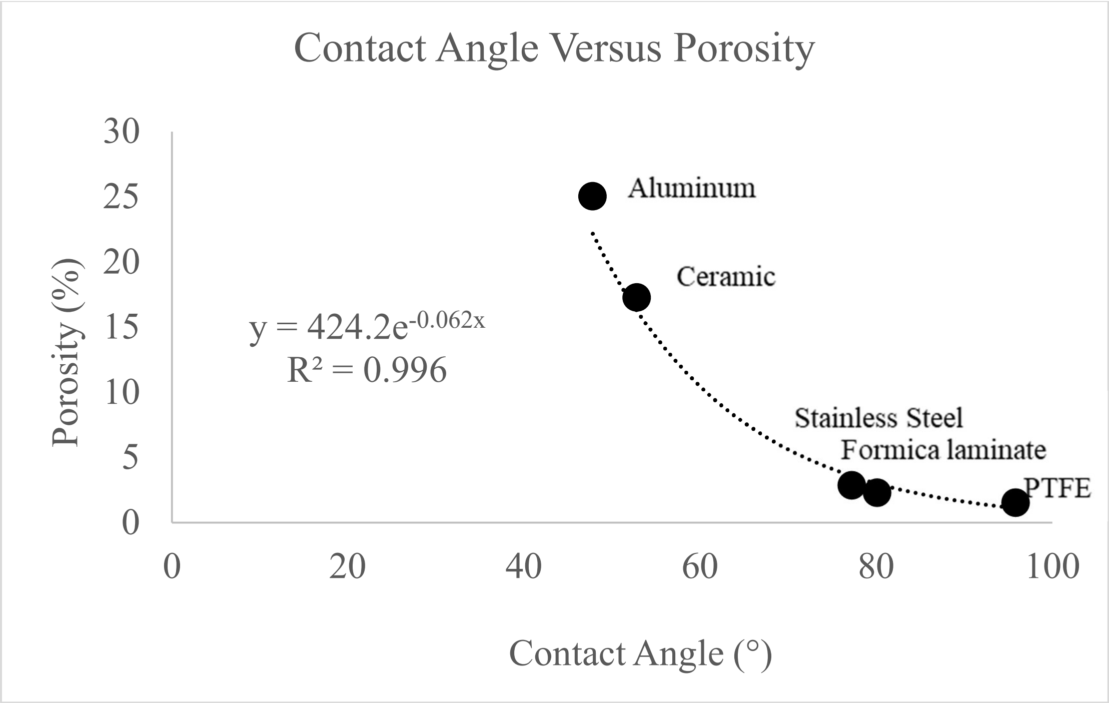

Figure S11. displays contact angle versus porosity for aluminum, ceramic, Formica laminate, PTFE, and stainless steel. Contact angle (°) is shown on the x axis. Percent surface porosity is shown on the y axis.

Figure S11 displays contact angle versus SEM porosity for aluminum, ceramic, Formica laminate, PTFE, and stainless steel. The surface characteristics of these five materials were quantified by our laboratory, however, BL_{405} experimentation was conducted on ceramic, PTFE, and stainless steel. These data display an inverse, exponential relationship, such that as contact angle (°) increases, percent porosity decreases.

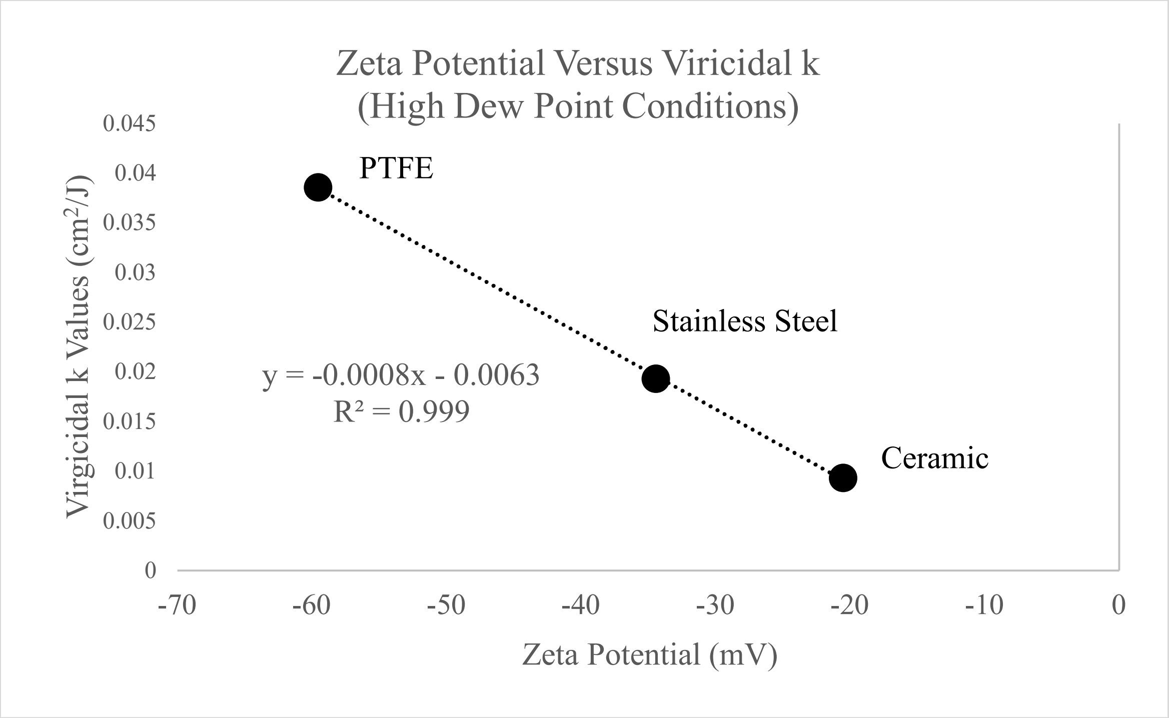

Figure S12. displays the zeta potential (mV) of PTFE, stainless steel, and ceramic versus the viricidal k values in the high dew point environments.

A strong inverse, linear correlation (R^2=0.999) between the zeta potential (mV) and viricidal k values of each material can be observed in Figure S10. This correlation exists for the viricidal k values calculated for each surface in the high dew point conditions. As zeta potential increases (becomes less negative), the viricidal k value decreases. The PTFE surface had the lowest and most negative zeta potential (mV), although had the highest viricidal k value. However, the ceramic surface had the highest zeta potential (mV), and the lowest viricidal k value in the high dew point conditions.

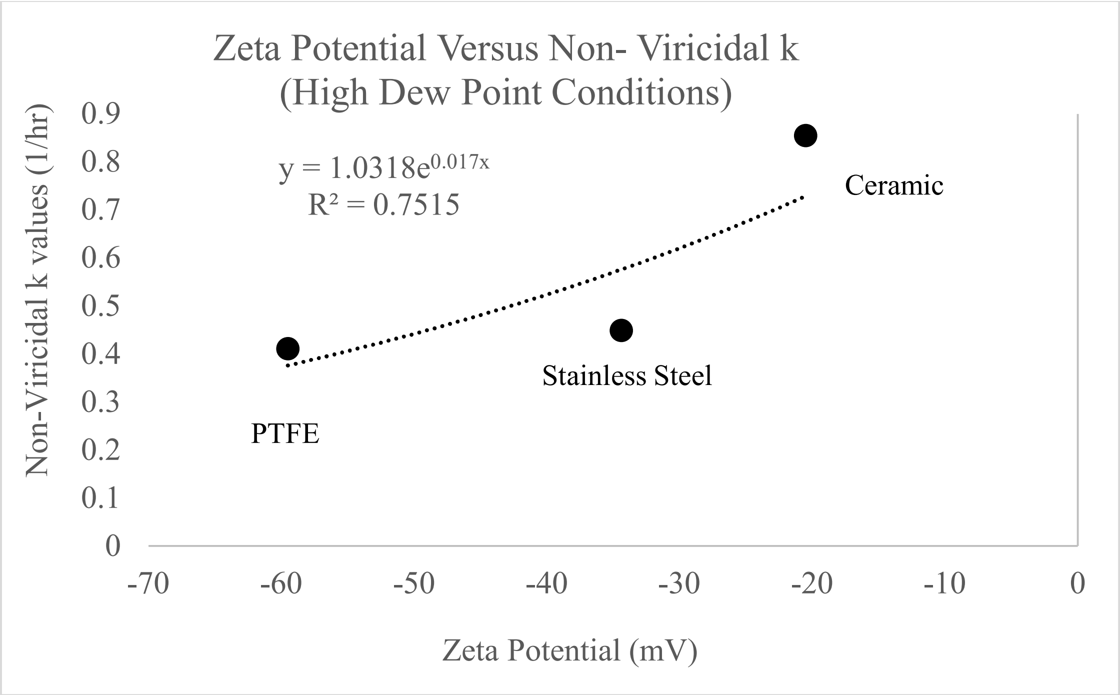

Figure S13. displays the zeta potential (mV) for PTFE, stainless steel, and ceramic as a function of the non-viricidal k values in the high dew point conditions.

A nonlinear relationship between zeta potential (mV) and non-viricidal k values for each surface is displayed in Figure S13. As zeta potential increases (becomes less negative), the non-viricidal k values increase. This correlation was observed only for the high dew point conditions. The PTFE surface had the lowest zeta potential (most negative) and non-viricidal k values. The ceramic surface had the highest zeta potential (mV) corresponding non-viricidal k values.
